# Isolation and Reactivity of an Antiaromatic s‐Block Metal Compound

**DOI:** 10.1002/anie.202014557

**Published:** 2020-12-27

**Authors:** Dipak Kumar Roy, Tobias Tröster, Felipe Fantuzzi, Rian D. Dewhurst, Carsten Lenczyk, Krzysztof Radacki, Conor Pranckevicius, Bernd Engels, Holger Braunschweig

**Affiliations:** ^1^ Institute for Inorganic Chemistry Julius-Maximilians-Universität Würzburg Am Hubland 97074 Würzburg Germany; ^2^ Institute for Sustainable Chemistry & Catalysis with Boron Julius-Maximilians-Universität Würzburg Am Hubland 97074 Würzburg Germany; ^3^ Discipline of Chemistry Indian Institute of Technology Indore Khandwa Road, Simrol Indore 453552, M.P. India; ^4^ Institute for Physical and Theoretical Chemistry Julius-Maximilians-Universität Würzburg Emil-Fischer-Strasse 42 97074 Würzburg Germany

**Keywords:** antiaromaticity, aromaticity, beryllium, heterocycles, s-block metals

## Abstract

The concepts of aromaticity and antiaromaticity have a long history, and countless demonstrations of these phenomena have been made with molecules based on elements from the p, d, and f blocks of the periodic table. In contrast, the limited oxidation‐state flexibility of the s‐block metals has long stood in the way of their participation in sophisticated π‐bonding arrangements, and truly antiaromatic systems containing s‐block metals are altogether absent or remain poorly defined. Using spectroscopic, structural, and computational techniques, we present herein the synthesis and authentication of a heterocyclic compound containing the alkaline earth metal beryllium that exhibits significant antiaromaticity, and detail its chemical reduction and Lewis‐base‐coordination chemistry.

## Graphical abstract

Using spectroscopic, structural, and quantum‐chemical methods, a heterocyclic compound containing the alkaline earth metal beryllium was characterized, which exhibits significant antiaromaticity. This species was subjected to chemical reduction and Lewis base coordination.

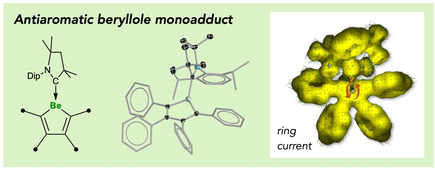

## Introduction

The concept of antiaromaticity in molecules was presented in the mid‐1960s by Breslow and co‐workers[^1^, ^2^] as a simple counterpoint to aromaticity, that is, aromatic compounds are those in which cyclic delocalization of electrons has a stabilizing effect, while cyclic delocalization imparts destabilization in antiaromatic compounds. The concept has since been expanded significantly in terms of our understanding of what constitutes antiaromaticity, the techniques available to measure it, and the number of isolated molecules fitting an antiaromatic description.[^3^, ^4^, ^5^] The currently accepted prerequisites for aromaticity and antiaromaticity are that the molecule must be cyclic, planar, and have an unbroken series of orbitals available for π bonding around the ring. From this point, the properties of aromatic and antiaromatic compounds diverge in a number of key ways, including:

- their stability (aromatics are more stable than their acyclic equivalents, while antiaromatics are less stable)
- the number of electrons in their π system (4*n*+2 π electrons for aromatics and 4*n* π electrons for antiaromatics)
- the magnetic ring current produced by delocalization (diamagnetic for aromatics and paramagnetic for antiaromatics)
- the bond‐length equilibration generally found in aromatics is not usually present in antiaromatics

Over the past few decades, the increasing ubiquity of two techniques has allowed more confident identification of antiaromatic species, namely single‐crystal X‐ray diffraction (for determination of planarity and bond lengths) and computational chemistry approaches, in particular nucleus‐independent chemical shift (NICS) methods,[^4^, ^5^] which determine the magnetic shielding at the center of a ring or above it. While truly antiaromatic hydrocarbons based on small ring systems are by definition unstable and thus very difficult to prepare and authenticate, the concept of antiaromaticity has found fertile ground in polycyclic π systems and heterocyclic chemistry, as is apparent from the proliferation of heteroatom‐containing antiaromatics such as porphyrins and porphyrinoids,[^6^, ^7^, ^8^, ^9^] boroles,[^10^, ^11^, ^12^, ^13^, ^14^, ^15^] and other boron‐containing heterocycles.^[16]^ Metals of the p, d, and f blocks of the periodic table have also proven able to participate in both aromaticity and antiaromaticity, as predicted theoretically[^17^, ^18^] and as confirmed by synthesis and structural authentication, exemplified by the now well‐represented families of alumoles,[^19^, ^20^, ^21^, ^22^] metallabenzenes,[^23^, ^24^, ^25^, ^26^, ^27^] and metallacyclopentadienes.[^28^, ^29^, ^30^] The extension of the aromaticity and antiaromaticity concepts to spherical systems,^[31]^ such as fullerenes,^[32]^ metallo‐fullerenes,^[33]^ molecular cages,[^34^, ^35^] and heterometallic clusters,[^36^, ^37^] is also attracting intense interest. This evidences the substantial importance of these concepts to the rationalization, design, and preparation of novel chemical structures.

In marked contrast to their p, d, and f block congeners, the metals of the s block of the periodic table are generally considered to be inflexible in terms of their oxidation states and have little ability to engage in π‐bonding interactions,^[38]^ which would seemingly disqualify them from forming either aromatic or antiaromatic compounds. However, over the last dozen years, work in s‐block chemistry has uncovered surprising oxidation‐state flexibility in the lighter Group 2 metals, including formally Mg^I^,[^39^, ^40^] Be^0^,^[41]^ and Be^I^ species.^[42]^ A number of s‐block species also exist that possess the six π electrons required for aromaticity, for example base‐stabilized 1,3‐diaza‐2‐berylloles and ‐magnesioles,^[43]^ although the aromaticity of these species was not discussed in the original articles. One report exists of the synthesis of molecules containing s‐block metals as part of 4π‐electron rings, namely [MgC_4_R^1^_2_R^2^_2_] (R^1^=SiMe_3_; R^2^=Me, Ph),^[44]^ and these were subsequently calculated to be significantly antiaromatic.^[45]^ However, these species were not structurally authenticated, preventing the crucial confirmation of their planarity. A complex of one of these magnesioles with the chelating ligand *N*,*N*,*N′,N′*‐tetramethyl‐1,2‐ethylenediamine (TMEDA) was structurally confirmed,^[44]^ but the tetracoordinate nature of the Mg atom in this complex suggests that a contiguous π network is not present in this compound. A method to construct an antiaromatic complex of beryllium has also recently been predicted by density functional theory (DFT) calculations,^[46]^ involving the conceptual combination of a butadiendiyl diradical [C_4_R_4_] fragment with the anionic fragment [:BeR]^−^ (R=anionic substituent), leading to an anionic 4π‐electron species. Nevertheless, despite extensive computational design, synthetic effort, and a number of promising results, the goal of preparing an antiaromatic s‐block complex remains out of reach.

Our approach to this problem was to conceptually combine the four‐π‐electron [C_4_R_4_] fragment with the neutral, zero‐π‐electron fragment [:BeL] (L=neutral Lewis donor), which would lead to a neutral species with four π electrons and thus circumvent the potential complications of the negatively charged products predicted theoretically.^[46]^ As the fragment [:BeL] is isoelectronic and isolobal with the boron‐containing fragment [:BR] (R=anionic substituent), present in the diverse family of antiaromatic boroles, we reasoned that this approach had a good chance of success. Interest in the chemistry of carbene‐stabilized beryllium species has exploded over the past few years, leading to the discovery of a range of Be^0^, Be^I^, and Be^II^ complexes with fascinating properties and reactivity, as well as providing a host of precursors containing stabilized [:BeL] scaffolds for the construction of compounds with unusual electronic structures.[^41^, ^42^, ^43^, ^47^] The [:BeL] fragment we chose was [:Be(CAAC)], bearing a neutral cyclic (alkyl)(amino)carbene (CAAC) donor, inspired by our previous synthesis of the precursor [(CAAC)BeCl_2_] and its use to form a compound in which the formally zerovalent Be atom takes part in strong multiple bonding with carbon, [Be(CAAC)_2_].^[41]^ Herein, we present the synthesis and isolation of a Lewis‐base‐stabilized beryllole, a heterocyclic organoberyllium compound, which was subsequently determined by spectroscopic, structural, and computational techniques to have significant antiaromaticity in its *cyclo*‐BeC_4_ ring. Its facile reactivity, including chemical reduction and the addition of a second Lewis donor, provides stable, non‐antiaromatic products.

## Results and Discussion

**Figure 1:**
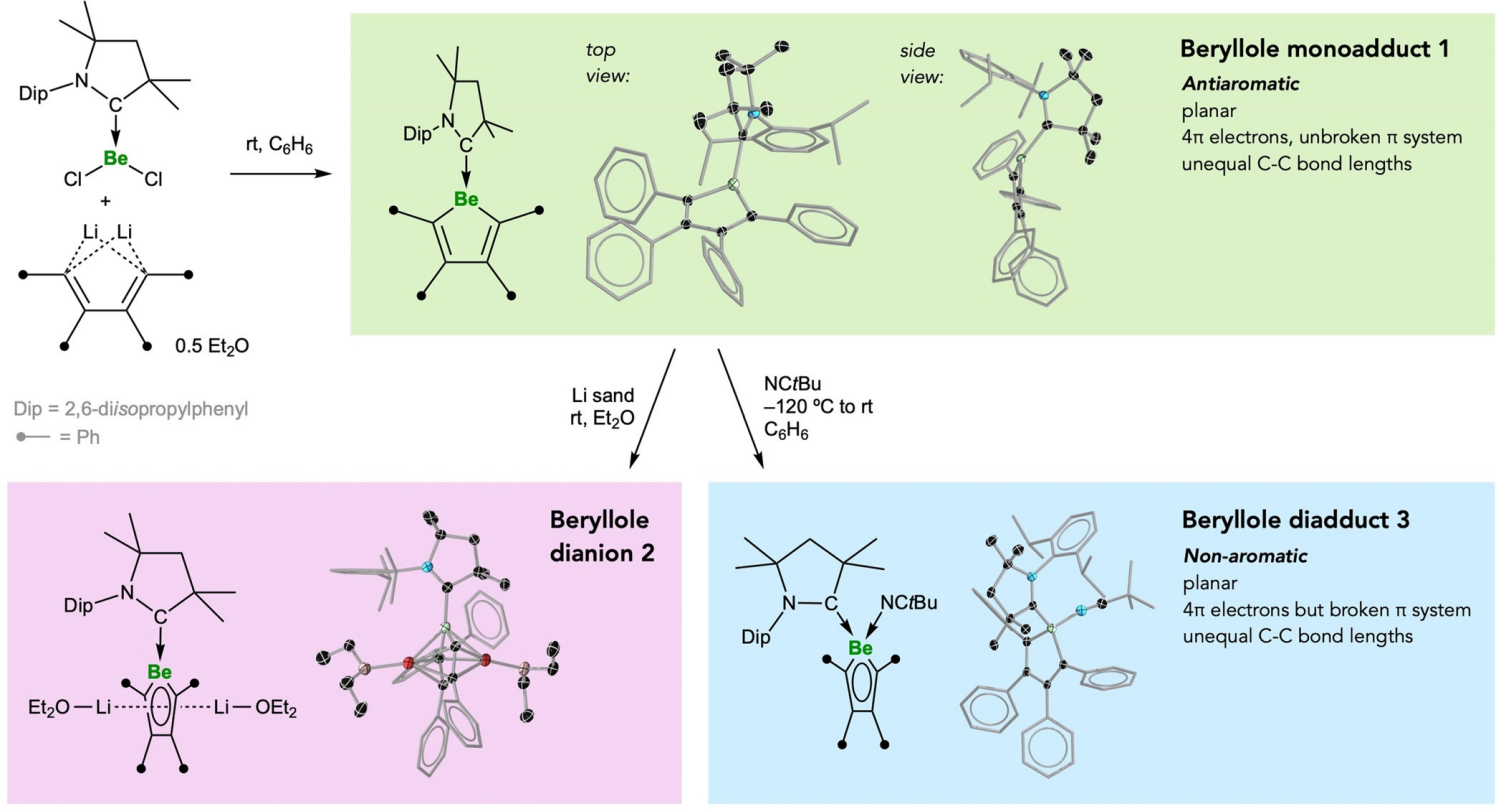
Synthesis and reactivity of the antiaromatic beryllole **1**, and solid‐state structures of **1‐3**.^[78]^ Ellipsoids of the crystallographically derived structures are shown at the 50 % probability level. All hydrogens and ellipsoids of peripheral groups have been removed for clarity. Atom colors: N blue, Be green, Li red, O pink.

**Synthesis and Reactivity of Beryllole 1**. Combination of [(CAAC)BeCl_2_] with dilithium tetraphenylbutadiene as its diethyl etherate (Li_2_[C_4_Ph_4_]⋅0.5 Et_2_O) in benzene at room temperature provided, after stirring and purification, an air‐sensitive yellow crystalline solid (yield 38 %) with a ^9^Be NMR spectroscopic shift at 22.9 ppm. Multinuclear NMR spectroscopy, high‐resolution mass spectrometry, and single‐crystal X‐ray diffraction techniques unambiguously identified this compound as the CAAC‐stabilized beryllole monoadduct [(CAAC)BeC_4_Ph_4_] (**1**) as shown in Figure 1. In order to place the ^9^Be NMR signal of **1** in context with comparable compounds, the related [(CAAC)BeCl_2_] showed a ^9^Be NMR signal at 12.9 ppm^[41]^ while that of the only known CAAC complex of a diorganyl beryllium, [(CAAC)Be(9,10‐anthracenyl)], was found at 1.7 ppm.^[47b]^ Both signals are significantly upfield of that of **1**, suggesting that the Be center in **1** is highly unsaturated, likely due to the low electronegativity of the attached sp^2^ carbon atoms of the C_4_ ring and the absence of delocalization of the four π electrons in this ring with the Be p orbital.

The solid‐state structure of **1** confirmed the tricoordinate nature of the Be center, the effective planarity of the BeC_4_ ring (deviations of the BeC_4_ atoms from the calculated least‐squares plane of this ring: 0.021–0.094 Å), and significant bond‐length alternation in the C_4_ backbone (inner C−C distance: 1.512(2) Å; outer C−C distances: 1.359(2), 1.358(2) Å). The planarity of the ring and bond‐length alternation in the backbone are both key structural attributes required for antiaromaticity, however, an analogous non‐aromatic species would also show the same attributes, for example, cyclopentadiene, which in one published solid‐state structure has an inner C−C distance of 1.460(1) Å and outer C−C distances of 1.344(1) Å in its butadiene backbone.^[48]^ Thus, the structural aspects of ring planarity and bond length alternation alone do not allow distinction between antiaromatic/nonaromatic descriptions.

Given the presumed antiaromaticity of beryllole monoadduct **1**, this compound was subjected to reactivity tests that parallel those previously demonstrated for the related family of antiaromatic boroles,[^10^, ^11^, ^12^, ^13^, ^14^, ^15^] namely two‐electron reduction and the addition of neutral, two‐electron donor groups (Lewis bases). Thereby, stirring **1** in a suspension of diethyl ether and lithium sand, followed by filtration, crystallization, and drying, provided a 50 % yield of an air‐ and thermally sensitive dark‐red crystalline solid determined to be the dilithium dietherate [Li(OEt_2_)]_2_[(CAAC)BeC_4_Ph_4_] (**2**, Figure 1) by multinuclear NMR spectroscopy, single‐crystal X‐ray diffraction, and elemental analysis. The solid‐state structure of **2** shows one [Li(OEt_2_)] fragment bound to each side of the BeC_4_ plane, bound somewhat more closely to the carbon atoms than the beryllium atom (avg. Li−C distance: 2.177 Å; avg. Li−Be distance: 2.358 Å). Compound **2** shows distinct bond distance equalization (inner C−C distance: 1.448(3) Å; outer C−C distances: 1.459(2), 1.470(2) Å), in line with the formally aromatic nature of the [(CAAC)BeC_4_Ph_4_]^2−^ heterocycle. However, the π coordination of two [Li(OEt_2_)]^+^ fragments to the ring precludes the description of **2** as a classical aromatic species.

Alternatively, treatment of beryllole monoadduct **1** with pivalonitrile (NC*t*Bu) at −120 °C, followed by warming to room temperature, evaporation of solvent and recrystallization, provided a 90 % yield of a yellow crystalline solid (**3**) with a ^9^Be NMR spectroscopic signal at 4.4 ppm. This signal is significantly upfield of that of **1** (22.9 ppm), reflective of the increase in electron density at Be upon quaternization (i.e. from six to eight valence electrons). The widths at half‐height (*ω*_1/2_) of reported ^9^Be NMR signals have recently been surveyed in relation to coordination number by Buchanan and Plieger,^[49]^ finding that these values decrease with increasing coordination number at Be due to its increased symmetry. Accordingly, the ^9^Be signal of tetracoordinate **3** is significantly less broad (*ω*_1/2_≈141 Hz) than the very broad signal of tricoordinate **1** (*ω*_1/2_≈506 Hz). However, this value for **3** is itself very broad for a tetracoordinate Be compound, being only the second such compound with a *ω*_1/2_ value above 100 according to the review of Buchanan and Plieger, an effect that could be attributed to its relatively low symmetry. Nevertheless, the ^9^Be NMR chemical shifts of **1** and **3** fall within the ranges reported in the aforementioned review for their respective coordination number.

Multinuclear NMR spectroscopy, high‐resolution mass spectrometry, and single‐crystal X‐ray diffraction techniques confirmed this species to be the beryllole diadduct [(*t*BuCN)(CAAC)BeC_4_Ph_4_] (**3**, Figure 1), with a tetracoordinate Be center and strongly alternating C_4_ backbone bond distances (inner C−C distance: 1.515(2) Å; outer C−C distances: 1.357(2), 1.357(2) Å), these distances being effectively identical to those of **1**. The exo‐ and endocyclic Be−C distances of **3** are only marginally longer than those of **1** and are presumably the result of the increased coordination number of the Be center.

**Computational Determination of the Antiaromaticity of Synthesized Berylloles**. Given the structural similarity of the beryllole rings of **1** and **3**, we turned to theory to provide a more concrete determination of the antiaromaticity/nonaromaticity of the compounds. Calculations using DFT, complete active space self‐consistent field (CASSCF),^[50]^ and n‐electron valence state second‐order perturbation theory (NEVPT2)[^51^, ^52^, ^53^] were performed in order to describe the molecular and electronic structures of the beryllium compounds obtained herein (see SI for details). The nature of the Be−CAAC bond in a truncated model of **1** was investigated using the energy decomposition analysis with natural orbitals for chemical valence (EDA‐NOCV) method.[^54^, ^55^] The antiaromatic character of the systems was investigated by NICS and aromatic stabilization energy (ASE) calculations,[^56^, ^57^] the latter obtained by the thermodynamic analysis of selected homodesmotic reactions.

**Figure 2:**
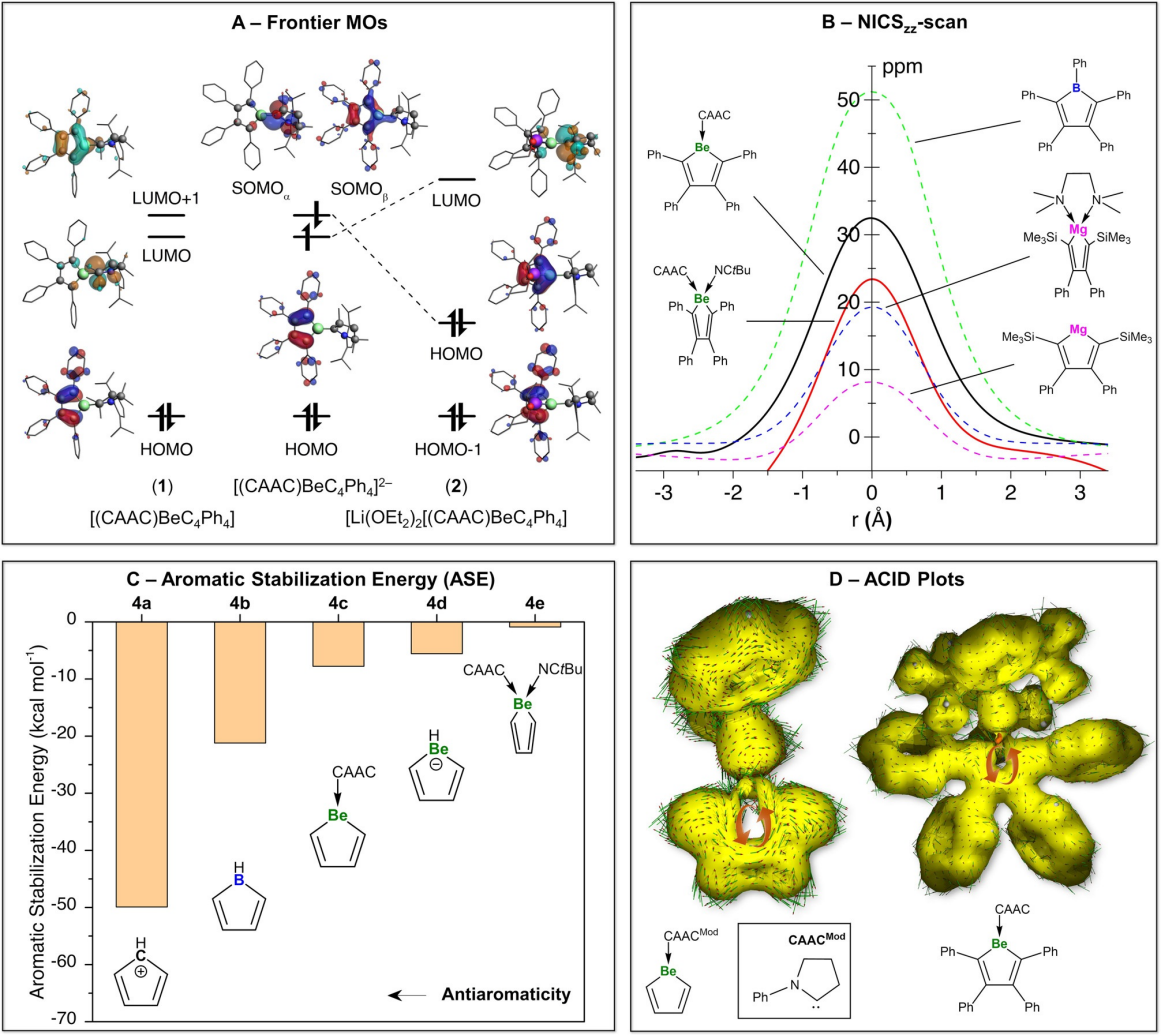
A) Frontier molecular orbitals, B) scan of the zz‐components of the nucleus independent chemical shifts (NICS_zz_‐scan), C) aromatic stabilization energies, and D) anisotropy of the induced current density (ACID) plots of selected (anti‐/non‐)aromatic compounds.

An informative initial picture of the bonding in these compounds can be obtained from appraisal of the frontier molecular orbitals (FMOs, Figure 2 A) of **1**, **2**, and those of a model compound where the [Li(OEt_2_)] fragments of **2** are removed, thus leading to the naked dianion [(CAAC)BeC_4_Ph_4_]^2−^. As expected, the HOMO of **1** (Figure 2 A, left) is very similar to that of the closely related antiaromatic borole species [PhBC_4_Ph_4_],[^58^, ^59^, ^60^] and is composed of an antisymmetric π orbital with the beryllium atom in the nodal position. A low‐lying virtual orbital of π symmetry in which the beryllium atom occupies a non‐nodal position, reminiscent of one of the degenerate HOMOs of the triplet cyclopentadienyl cation, C_5_H_5_^+^ (X ^3^A_2_′ state of *D*_5*h*_ point group symmetry),[^61^, ^62^, ^63^] was also observed. However, while for boroles this orbital is the LUMO, for **1** it is the LUMO+1, as the LUMO of **1** is predominantly located on the CAAC ligand and can be described as predominantly π*(CN) in character. Thus, substitution of :BPh with the isolobal :Be(CAAC) moiety affects the FMOs of **1** mainly by introducing a second low‐lying vacant orbital, which, as discussed below, leads to remarkable consequences for the electronic structure of the beryllole dianion.

Computational addition of two electrons to boroles [R′BC_4_R_4_] results in the formation of borolyl dianions [R′BC_4_R_4_]^2−^. These 6π‐electron systems, although calculated to be aromatic and analogous to the aromatic cyclopentadienyl anion, C_5_H_5_^−^, are unknown in the absence of π‐coordinated s‐block metals. In contrast, analogous computational addition of two electrons to the CAAC‐stabilized beryllole **2** leads to the dianion [(CAAC)BeC_4_Ph_4_]^2−^ (Figure 2 A, middle), which, although possessing six π electrons, is singlet biradicaloid in nature, wherein the low‐lying LUMO and LUMO+1 of **1** are now partially occupied and the C−C bond lengths in the five‐membered ring are unequal. This picture is corroborated by DFT in the unrestricted formalism as well as by high‐level CASSCF/NEVPT2 calculations, the latter indicating a biradical character of 37.4 % for [(CAAC)BeC_4_Ph_4_]^2−^, which is of the same order as that of the recently isolated parent aluminene [(CAAC)_2_AlH],^[64]^ and a singlet‐triplet gap (Δ_T–S_) of 3.3 kcal mol^−1^. Interestingly, the presence of Li atoms bound to the five‐membered beryllole ring in compound **2** significantly stabilizes the low‐lying π* orbital associated with the ring, while that of the CAAC ligand is pushed to higher energies (Figure 2 A, right). As a result, in the experimentally realized species **2**, the 6π‐electron cloud is stabilized by the addition of two [Li(OEt_2_)]^+^ fragments, leading to a closed‐shell singlet analogous to an aromatic borole dianion, as well as C−C bond length equalization. Accordingly, the singlet–triplet gap for this system is 42.1 kcal mol^−1^ as obtained from CASSSCF/NEVPT2 calculations.

The aforementioned NICS technique is a useful computational gauge of the magnetic shielding at a given point near a ring system, allowing a measure of aromaticity, antiaromaticity, and non‐aromaticity of ring systems.[^4^, ^5^] In order to obtain more reliable and comparable data, the so‐called zz component of the magnetic shielding tensor (NICS_zz_) is commonly reported in the literature.[^65^, ^66^] The NICS_zz_ values of the experimentally realized compounds **1** and **3** were calculated, along with a number of other relevant molecules for comparison, starting at the ring centroid and at 0.1 Å steps on an axis perpendicular to the ring plane (in both directions), leading to NICS_zz_‐scan curves.[^67^, ^68^] These data, obtained at the B3LYP[^69^, ^70^, ^71^, ^72^]/6‐311++G**[^73^, ^74^] level of theory, are displayed in Figure 2 B. A large negative NICS_zz_ value at a point 1 Å above and below the ring plane (denoted NICS_zz_(1) and NICS_zz_(−1)) is regarded as a hallmark of aromaticity, while rings with large positive NICS_zz_(1/−1) values can be regarded as antiaromatic. Accordingly, the NICS_zz_(1) value of the known antiaromatic species pentaphenylborole [PhBC_4_Ph_4_] was calculated to be +26.1 ppm. Our calculations show that the 4π‐electron beryllole monoadduct [(CAAC)BeC_4_Ph_4_] (**1**; NICS_zz_(1): +14.1/+13.5 ppm) is indeed antiaromatic, while the diadduct [(*t*BuCN)(CAAC)BeC_4_Ph_4_] (**3**; NICS_zz_(1): +5.3/+3.7 ppm), the π network of which is interrupted by the presence of a second donor ligand on the Be atom, is effectively nonaromatic. By comparison, the proposed magnesiole species [MgC_4_Ph_2_(SiMe_3_)_2_] (NICS_zz_(1): +0.8/ +0.7 ppm) is also nonaromatic, while its structurally authenticated diamine adduct [(TMEDA)MgC_4_Ph_2_(SiMe_3_)_2_] (NICS_zz_(1): +6.2/+6.0 ppm) could be regarded as very weakly antiaromatic.^[44]^

A number of further points should be noted in order to confirm the antiaromaticity of beryllole monoadduct **1** and distinguish it from beryllole diadduct **3**. The evident perpendicularity of CAAC and BeC_4_ planes of **1** (angle between planes: 79.5° in the solid‐state structure and 77.3° in the calculated structure) implies the absence of an exocyclic Be−C π interaction and that the CAAC unit acts as a pure σ‐donor. This picture is fully corroborated by the EDA‐NOCV calculations (see SI for details), which revealed the predominance of a donor–acceptor rather than an electron‐sharing Be−C^CAAC^ bond description in **1**, with negligible π interaction. Furthermore, computationally reducing the size of the BeC_4_ backbone substituents of **1** causes the tilt of the CAAC unit out of the BeC_4_ plane to approach zero (calculated tilt angles for [(CAAC)BeC_4_Ph_4_]: 148.0°; [(CAAC)BeC_4_Me_4_]: 160.9°; [(^H^CAAC)BeC_4_H_4_]: 179.8°; ^H^CAAC is the hypothetical unsubstituted CAAC ligand pyrrolidin‐2‐ylidene (C_4_H_7_N), see SI), suggesting that the tilting observed in the solid‐state structure is caused by steric, rather than electronic, effects.

In order to test the hypothesis that the presence of four electrons in the BeC_4_ π system of **1** is more unfavorable than two analogous molecules containing two π electrons each, we calculated aromatic stabilization energies (ASEs) of a range of related five‐membered C_4_H_4_X rings (Figure 2 C). These systems contain four π electrons each, and their ASEs were calculated following the homodesmotic reaction depicted in Equation 1.[^56^, ^57^]
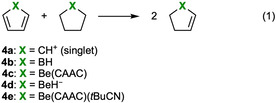



The zero‐point‐energy‐corrected electronic energies of the reactions depicted in Equation (1) were calculated using the molecules in their most stable conformations, and thus it is expected that contributions coming from strain effects are suppressed. The ASEs following Equation (1) are thus directly associated with cyclic delocalization/localization effects. Positive ASE values are related to the stability of aromatic compounds due to cyclic delocalization, whereas negative ASE values denote destabilization of antiaromatic species via cyclic localization. The singlet cyclopentadienyl cation C_5_H_5_^+^ (**4 a**, ^1^A_1_ state of *C*_2*v*_ point group symmetry), which is a low‐lying excited state above the X ^3^A_2_′ ground state,^[64]^ has the largest destabilization energy (ASE=−49.9 kcal mol^−1^), and therefore is the most antiaromatic C_4_H_4_X ring among those studied herein. This is followed by the borole ring **4 b**, with an ASE value of −21.2 kcal mol^−1^. The beryllium‐containing systems **4 c** and **4 d** also present negative ASE values (−7.8 and −5.6 kcal mol^−1^, respectively), thus confirming the destabilizing thermodynamic effect and further supporting the antiaromatic description of **1**. Finally, calculations for the reduced model of the beryllole diadduct **4 e** found an ASE value of practically zero (ASE=−0.9 kcal mol^−1^), thus confirming the nominal antiaromaticity/non‐aromaticity of this species.

Finally, we probed the antiaromaticity of CAAC‐stabilized BeC_4_ rings by using the anisotropy of the induced current density (ACID) method, as proposed by Herges and co‐workers.[^75^, ^76^] ACID has been widely used for assessing delocalization, conjugation, and aromaticity in distinct molecular systems.[^76^, ^77^] Briefly, by plotting the ACID scalar field isosurfaces together with the current density vectors, it is possible to distinguish between diatropic (clockwise) and paratropic (counterclockwise) π‐electron circulation, which are characteristic of aromatic and antiaromatic molecules, respectively. According to the ACID analysis (Figure 2 D), BeC_4_ rings exhibit counterclockwise, paratropic circulation typical of antiaromatic systems. Furthermore, the heteroatom contribution to the cyclic conjugation is lower in berylloles than boroles (see Figure S15), suggesting that the antiaromatic character of the studied berylloles is less pronounced than that of boroles. These results are consistent with those found using the aromatic stabilization energy and NICS calculations, supporting our attribution of berylloles as species with distinct but weak antiaromatic character. Future work in our group will include a detailed investigation of the electronic structure of the dilithio beryllole **2** and the electrostructural effects dictating the biradicaloid character of its naked form [(CAAC)BeC_4_Ph_4_]^2−^.

**Figure 3:**
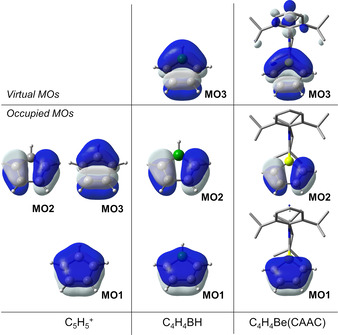
Relevant molecular orbitals calculated for truncated cyclic systems [XC_4_H_4_] (X=[HC]^+^, to [HB], [(CAAC)Be]), highlighting the extent of participation of the X fragment in the ring π system.

Our results indicate a dramatic decrease in the antiaromaticity in the ring systems [XC_4_H_4_] upon moving left from carbon in the periodic table, that is, from X=[HC]^+^, to [HB], and to [(CAAC)Be]. The reason for this decrease can be understood by considering the interaction of the π‐MOs of the fragments C_4_H_4_ and X. Figure S16 provides a sketch of the variations obtained for different X fragments while Figure 3 compares the computed MOs of the systems. For clarity we only focus on the MOs of relevance to the XC_4_H_4_ π system. Upon varying X, the relative energy of the MO in which X lies on the nodal plane (MO2) remains nearly unchanged, but the energy of MO3, to which X contributes, changes its position. For X=CH this orbital is degenerate with MO2. The energy of this orbital is higher for X=[HB] and [(CAAC)Be] because the fragment orbital of X increases in energy, thereby lifting the degeneracy. Furthermore, the mixing of the fragment orbitals decreases because the energy difference between the fragment orbitals (i.e. C_4_H_4_ vs. X) increases. As a consequence, the mixing also decreases so that the fragments become increasingly decoupled. This decoupling is best seen in MO1 (Figure 3), which for X=[HC]^+^ is completely delocalized, but for X=[(CAAC)Be] is more localized on the [C_4_H_4_] fragment. This decoupling in the beryllole system leads to its reduced antiaromaticity.

## Conclusion

The structural, thermodynamic, and magnetic considerations detailed above indicate that the beryllole monoadduct **1** has distinct antiaromaticity, setting it apart from the negligible antiaromaticity of the beryllole diadduct **3**. The results herein present the full spectroscopic, structural, and computational authentication of a true antiaromatic species based on the alkaline earth metal beryllium. The results suggest levels of oxidation‐state flexibility and participation in sophisticated π systems that are unprecedented for the metals of the s block, and hint at the potentially rich chemistry of molecules combining π systems and alkaline earth metals.

## Conflict of interest

The authors declare no conflict of interest.

## Supporting information

As a service to our authors and readers, this journal provides supporting information supplied by the authors. Such materials are peer reviewed and may be re‐organized for online delivery, but are not copy‐edited or typeset. Technical support issues arising from supporting information (other than missing files) should be addressed to the authors.

Supplementary
