# Supplementary material for: Isolation and Reactivity of an Antiaromatic s‐Block Metal Compound
Source: Angew Chem Int Ed Engl. 2020 Dec 27;60(7):3812–9. doi: 10.1002/anie.202014557 (PMC7898526; doi:10.1002/anie.202014557)
Supplement: Supplementary file 1 — Supplementary [file ANIE-60-3812-s001.pdf]

Angewandte Chemie
Eine Zeitschrift der Gesellschaft Deutscher Chemiker

Supporting Information

Isolation and Reactivity of an Antiaromatic s-Block Metal Compound

Dipak Kumar Roy, Tobias Tröster, Felipe Fantuzzi, Rian D. Dewhurst, Carsten Lenczyk, Krzysztof Radacki, Conor Pranckevicius, Bernd Engels, and Holger Braunschweig**

anie_202014557_sm_miscellaneous_information.pdf

Supplementary information

Table of Contents

Synthetic Details	S2
Crystallographic Details	S15
Computational Details	S19
References	S67

Synthetic Details

General synthetic considerations. All reactions were performed under an atmosphere of dry argon using standard Schlenk or glovebox techniques. $BeCl_2$ was purchased from Sigma-Aldrich and used as received. All solvents were purified by distillation using the appropriate drying agents, deoxygenated using three freeze–pump–thaw cycles and stored over molecular sieves under dry argon prior to use. The deuterated solvents used for NMR spectroscopy were purchased from Cambridge Isotope Laboratories, deoxygenated by freeze–pump–thaw cycles and dried under an argon atmosphere over molecular sieves. The following compounds were synthesized according to literature: CAAC,[1,2] dilithium tetraphenylbutadiene.[3] NMR chemical shifts are reported in ppm and coupling constants in Hz. ^{1}H, ^{9}Be and ^{13}C NMR spectroscopy data were obtained at ambient temperature using either a Bruker DRX-400 (operating at 400 MHz for ^{1}H, 56 MHz for ^{9}Be and 100 MHz for ^{13}C) or a Bruker Avance 500 NMR spectrometer (operating at 500 MHz for ^{1}H and 125 MHz for ^{13}C). ^{1}H NMR spectra were referenced via residual proton resonances of C_6D_6 (^{1}H, 7.16 ppm) and THF-d_8 (^{1}H, 3.58, 1.72 ppm). ^{13}C NMR spectra were referenced to C_6D_6 (^{13}C, 128.06 ppm), THF-d_8 (^{13}C, 67.21, 25.31 ppm). Electron paramagnetic resonance (EPR) measurements at the X band (9.38 GHz) were carried out at room temperature and at 70 K using a Bruker ELEXSYS E580 EPR spectrometer equipped with an Oxford Instruments helium cryostat (ESR 900) and a MercuryiTC temperature controller.

Synthesis of beryllole [(CAAC)BeC$_4$Ph$_4$] (1). A suspension of dilithium tetraphenylbutadiene (0.5 diethyletherate) (1.45 g, 3.56 mmol, 1.3 equiv) in benzene (10 mL) was treated with [(CAAC)BeCl$_2$] (1.00 g, 2.74 mmol, 1.0 equiv) and stirred at room temperature for 16 h. The solvent of the reaction mixture was removed *in vacuo*. The resulting solid was extracted with hexane (3 x 10 mL) and the filtrate was dried *in vacuo*. The resulting solid was recrystallized from diethyl ether, affording **1** as a yellow crystalline solid (680 mg, 38%). **^{1}H NMR** (C_6D_6, 500 MHz): δ 7.13-7.09 (m, 4H, CH_{Aryl}), 7.04-6.98 (m, 5H, CH_{Aryl}), 6.96-6.90 (m, 2H, CH_{Aryl}), 6.90-6.80 (m, 10H, CH_{Aryl}), 6.73-6.67 (m, 2H, CH_{Aryl}), 2.88 (sept, $^3J_{HH}$ = 6.7 Hz, 2H, CH_{Dipp}), 1.27 (s, 3H, CH_2), 1.18 (s, 6H, CH_3), 1.10-1.07 (m, 12H, CH_3), 0.93 (s, 6H, CH_3). **^{9}Be NMR** (C_6D_6, 56 MHz): δ 22.9 (very broad s, $\omega_{½}$ = ca. 506 Hz). **^{13}C{^{1}H} NMR** (C_6D_6, 125 MHz): δ 250.0 ($C_{qCarbene}$, detected by HMBC only), 161.2 (C_q), 152.6 (C_q), 145.3 (C_q), 143.3 (C_q), 134.4 (C_q), 130.4 (C_{Aryl}), 129.8 (C_{Aryl}), 127.9 (C_{Aryl}), 127.5 (C_{Aryl}), 127.0 (C_{Aryl}), 125.5 (C_{Aryl}), 124.7 (C_{Aryl}), 122.2 (C_{Aryl}), 82.5 (C_q), 56.5 (C_q), 49.6 (CH_2), 29.4 (CH), 29.3 (CH_3), 29.0 (CH_3) 25.3 (CH_3). **HRMS (LIFDI):** calculated for $C_{48}H_{51}BeN$ (M^+): 650.4138; found 650.4128.

Synthesis of dilithium berylloldiide $[Li(OEt_2)]_2[(CAAC)BeC_4Ph_4]$ (2). 1 (200 mg, 307 μmol, 1.0 equiv) was dissolved in diethyl ether (6 mL), treated with lithium sand (4.9 mg, 710 μmol, 2.3 equiv) and stirred for 2 h at room temperature. The reaction mixture was filtered. The filtrate was stored at –33 °C, affording **2** as a dark red crystalline solid (128 mg, 50%) via slow evaporation of the solvent. **^{1}H NMR** (Tol-d_8, 500 MHz): δ 7.00-6.99 (m, 1H, CH_{Aryl}), 6.99-6.98 (2H, m, CH_{Aryl}), 6.96-6.93 (8H, m, CH_{Aryl}), 6.92-6.90 (2H, m, CH_{Aryl}), 6.88-6.83 (m, 6H, CH_{Aryl}), 6.68 (m, 2H, CH_{Aryl}), 3.18-3.09 (m, 10H, CH_{Dipp} + C$H_{2\,Ether}$), 1.65 (s, 8H, CH_2 + CH_3), 1.19 (d, 6H, ^3J = 6.47 Hz, C$H_{3\,Dipp}$), 1.05 (s, 6H, CH_3), 0.97 (d, 6H, ^3J = 6.47 Hz, C$H_{3\,Dipp}$), 0.85 (t, 12H, ^3J = 7.09 Hz, C$H_{3\,Ether}$). **^{7}Li NMR** (Tol-d_8, 194 MHz): δ –4.00 (s). No signal was observed in ^{9}Be NMR spectra despite multiple attempts. **^{13}C{^{1}H} NMR** (Tol-d_8, 125 MHz): δ 156.3 (C_q), 147.30 (C_q), 145.6 (C_q), 136.3 (C_q), 133.5 (C_{Aryl}), 130.8 (C_{Aryl}), 129.1 (C_{Aryl}), 126.4 (C_{Aryl}), 126.0 (C_{Aryl}), 122.4 (C_{Aryl}), 121.6 (C_{Aryl}), 116.8 (C_{Aryl}), 80.74 (C_q), 65.4 ($CH_{2\,Ether}$), 56.7 (C_q), 51.4 (CH$_2$), 30.9 (CH$_3$), 29.6 (CH_{Dipp}), 29.4 (CH$_3$), 27.2 ($CH_{3\,Dipp}$), 24.0 ($CH_{3\,Dipp}$), 14.6 ($CH_{3\,Ether}$). **Mass spectra** could not be obtained for **2** due to decomposition. **Elemental analysis:** Calculated for $C_{56}H_{71}BeLiNO_2$: C 82.72, H 8.80, N 1.72; found C 81.17, H 8.57, N 1.71.

Synthesis of beryllole NC*t*Bu adduct [(*t*BuCN)(CAAC)BeC_4Ph_4] (3). 1 (20.0 mg, 30.7 μmol, 1.0 equiv) was dissolved in benzene (0.6 mL), frozen (−120 ºC), and pivalonitrile (3.83 mg, 5.09 μmol, 1.0 equiv) was added. The reaction mixture was allowed to warm to room temperature. After 1 h all volatiles were removed *in vacuo*. The resulting solid was recrystallized from a benzene/hexane mixture at room temperature, affording **3** as a yellow crystalline solid (20.3 mg, 90%). **^{1}H NMR** (C_6D_6, 500 MHz): δ = 7.21-7.20 (m, 2H, CH_{aryl}), 7.20-7.18 (m, 2H, CH_{aryl}), 7.15-7.14 (m, 2H, CH_{aryl}), 7.14-7.11 (m, 5H, CH_{aryl}), 7.11-7.10 (m, 1H, CH_{aryl}), 6.97- 6.95 (m, 1H, CH_{aryl}), 6.95- 6.94 (m, 2H, CH_{aryl}), 6.94- 6.92 (m, 2H, CH_{aryl}), 6.90-6.87 (m, 2H, CH_{aryl}), 6.81-6.77 (m, 4H, CH_{aryl}), 2.83 (sept, 2H, ^3J = 6.77 Hz, C*H*), 1.93 (s, 6H, CH_3), 1.65 (s, 2H, CH_2), 1.01 (s, 3H, CH_3), 1.00 (s, 3H, CH_3) 0.92 (s, 6H, CH_3), 0.64-0.62 (m, 15*H*, CH$_3$) ppm. **^{9}Be NMR** (C_6D_6, 56 MHz): δ = 4.4 (broad s, $\omega_{½}$ = ca. 141 Hz) ppm. **^{13}C{^{1}H} NMR** (C_6D_6, 125 MHz): δ 155.3 (C_q), 155.1 (C_q), 146.9 (C_q), 144.8 (C_q), 135.4 (C_q), 131.1 (C_{Aryl}), 126.8 (C_{Aryl}), 126.7 (C_{Aryl}), 124.5 (C_{Aryl}), 123.7 (C_{Aryl}), 121.0 (C_{Aryl}), 79.8 (C_q), 57.6 (C_q), 50.7 (CH$_2$), 29.9 (CH$_3$), 29.1 (CH$_3$), 29.0 (CH_{Dipp}), 27.9 (C_q), 27.3 (CH$_3$), 26.2 (CH$_3$), 23.1 (CH$_3$). **HRMS (LIFDI):** calculated for $C_{48}H_{51}BeN$ ([M – NC*t*Bu]$^+$): 650.4138; found 650.4125.

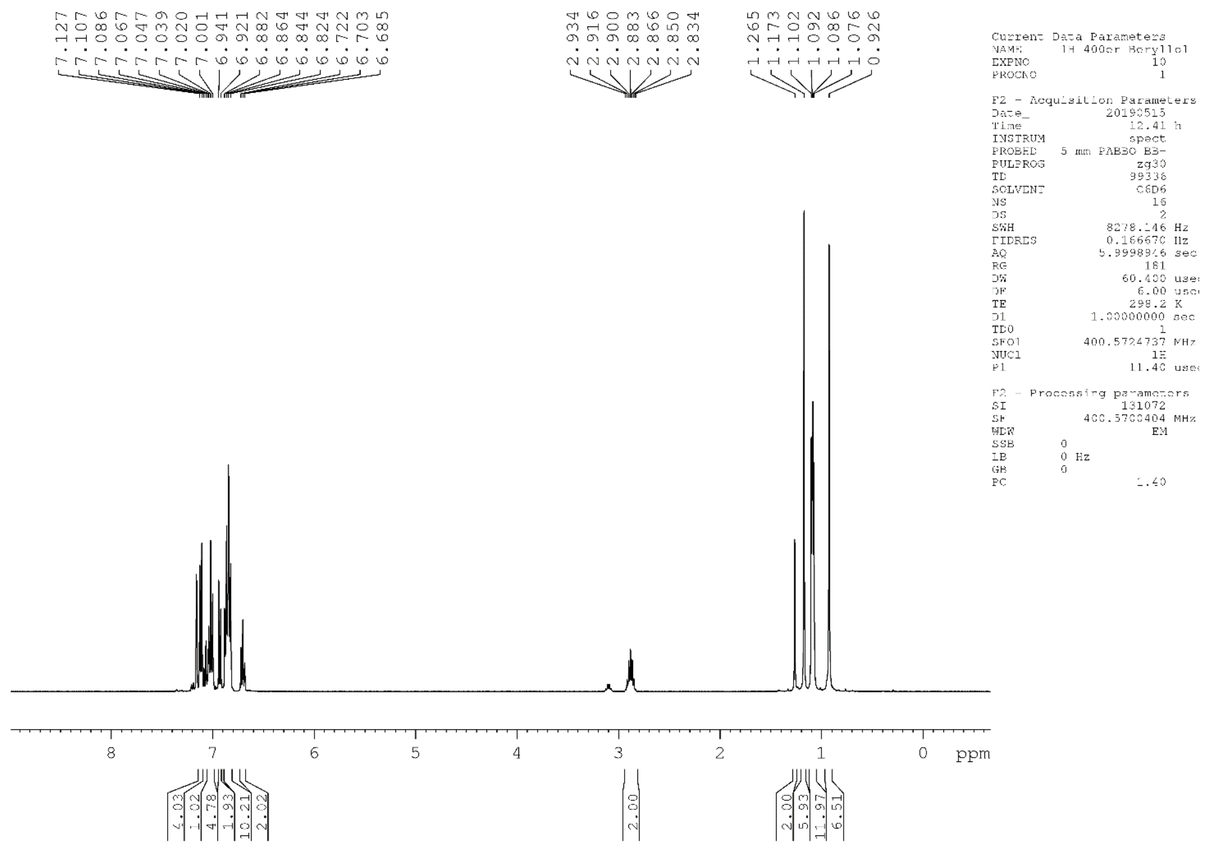

Figure S1. ^{1}H NMR spectrum of **1**.

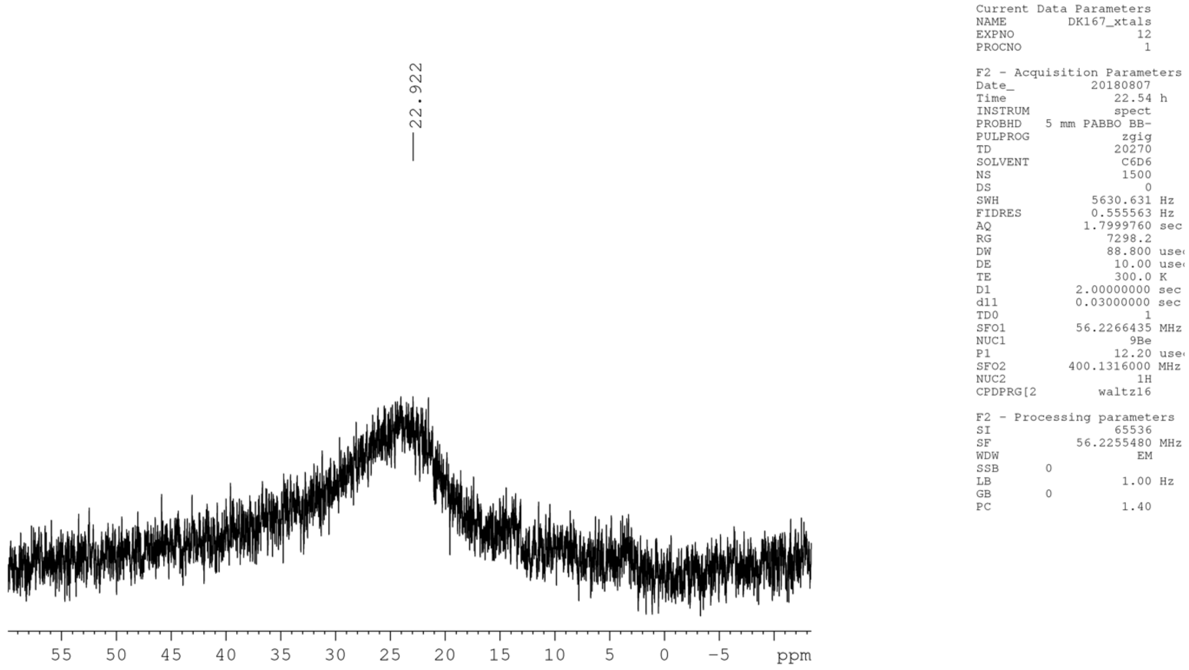

Figure S2. ^{9}Be NMR spectrum of **1**.

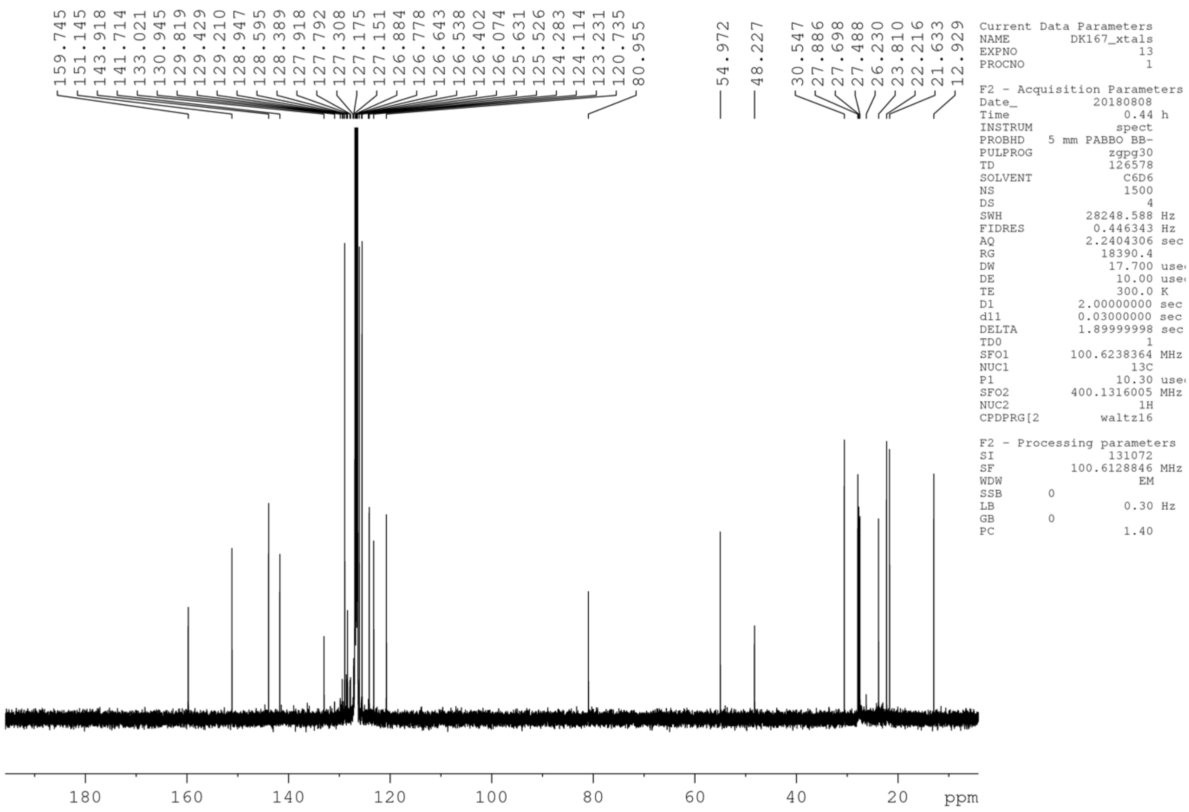

Figure S3. $^{13}C\{^{1}H\}$ NMR spectrum of **1**.

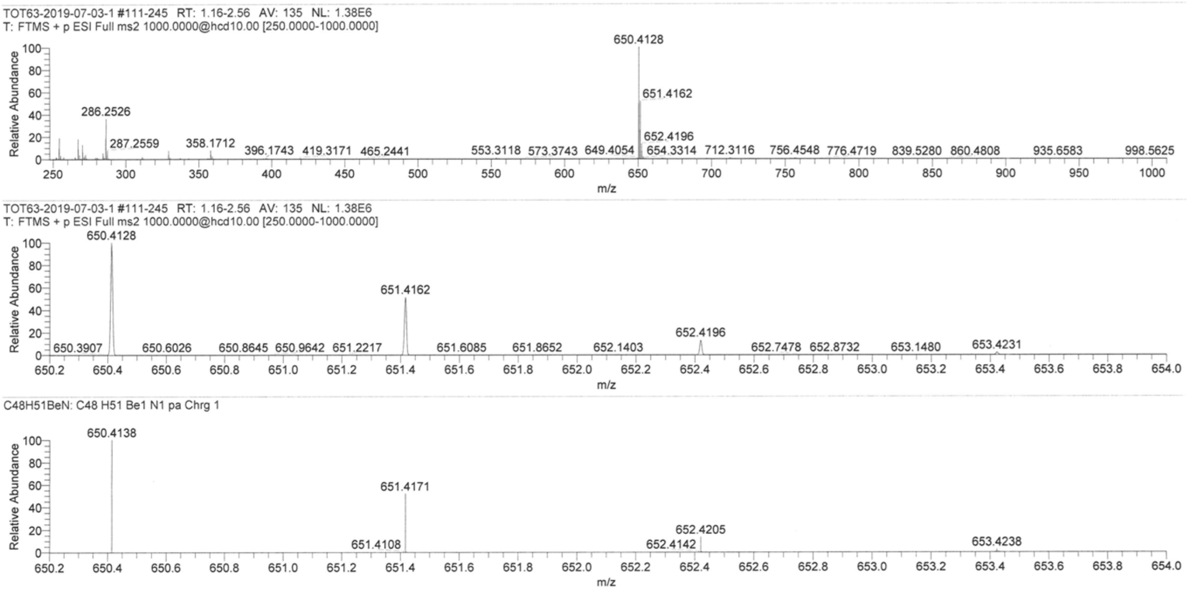

Figure S4. High-resolution LIFDI mass spectrum of **1** (top and middle). Calculated isotope pattern for $[M]^+$ (bottom).

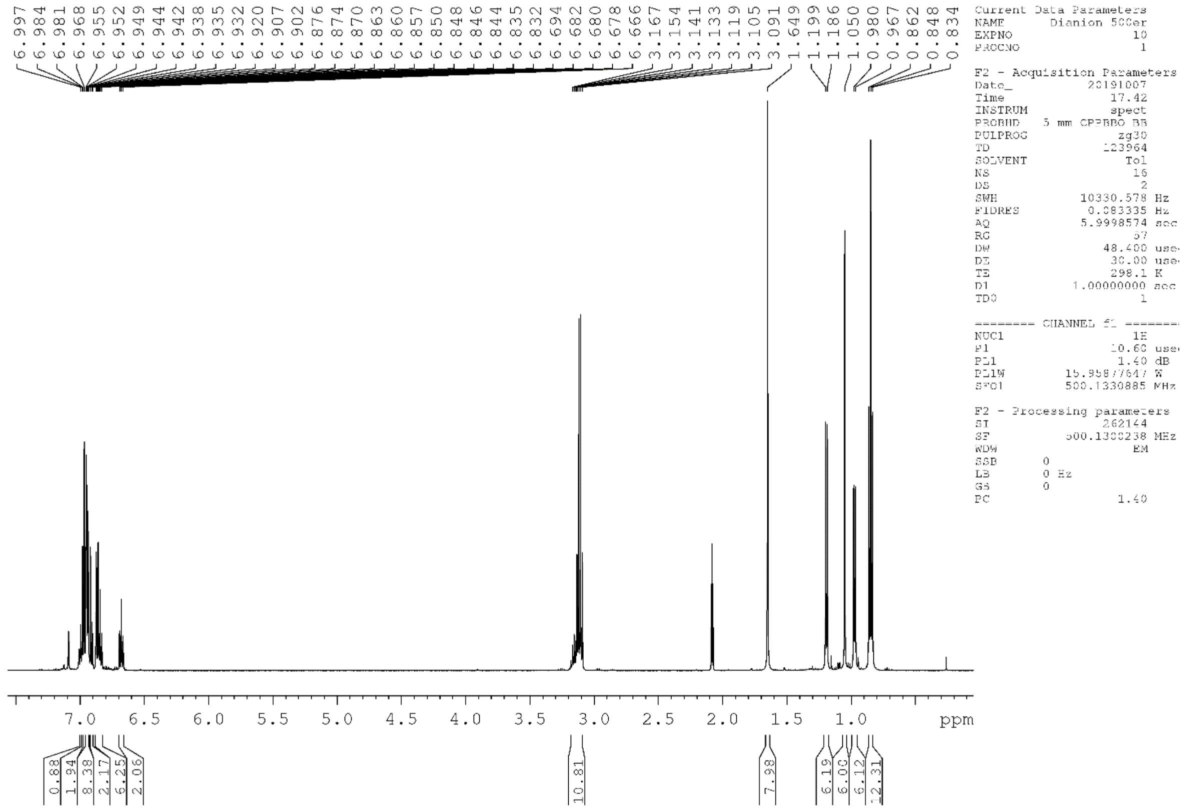

Figure S5. ^{1}H NMR spectrum of **2**.

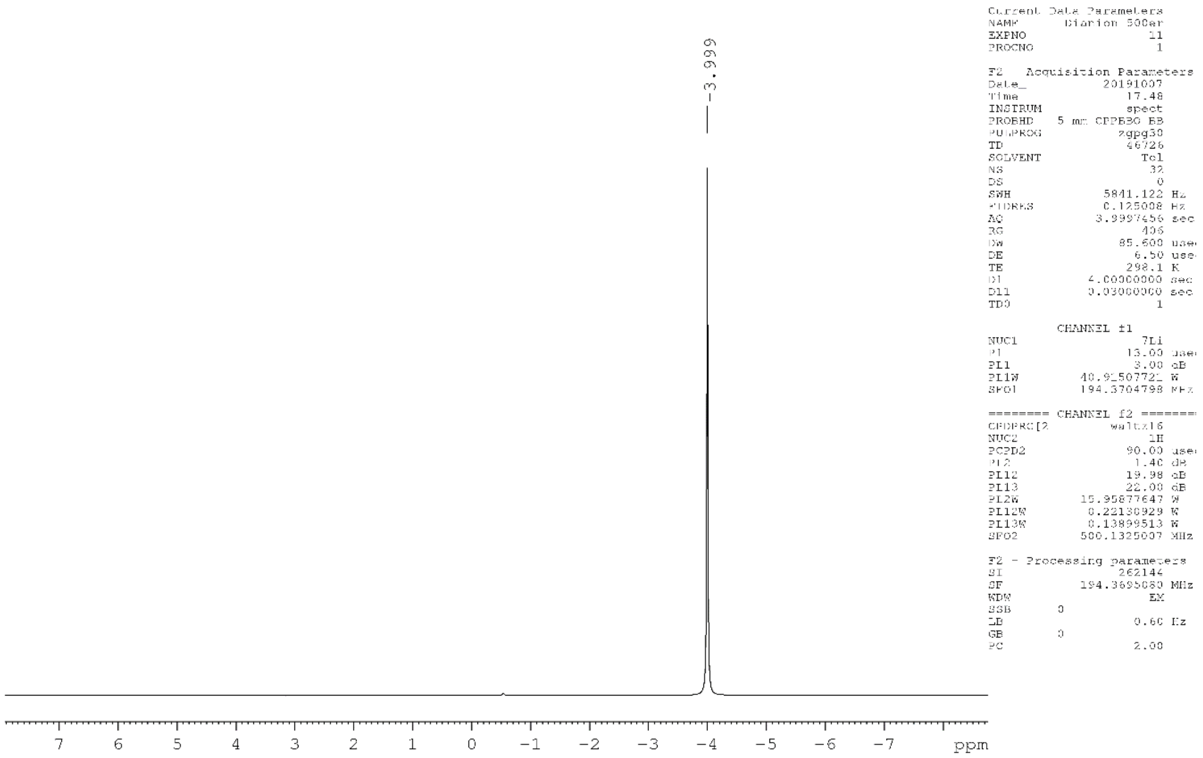

Figure S6. ^{7}Li NMR spectrum of **2**.

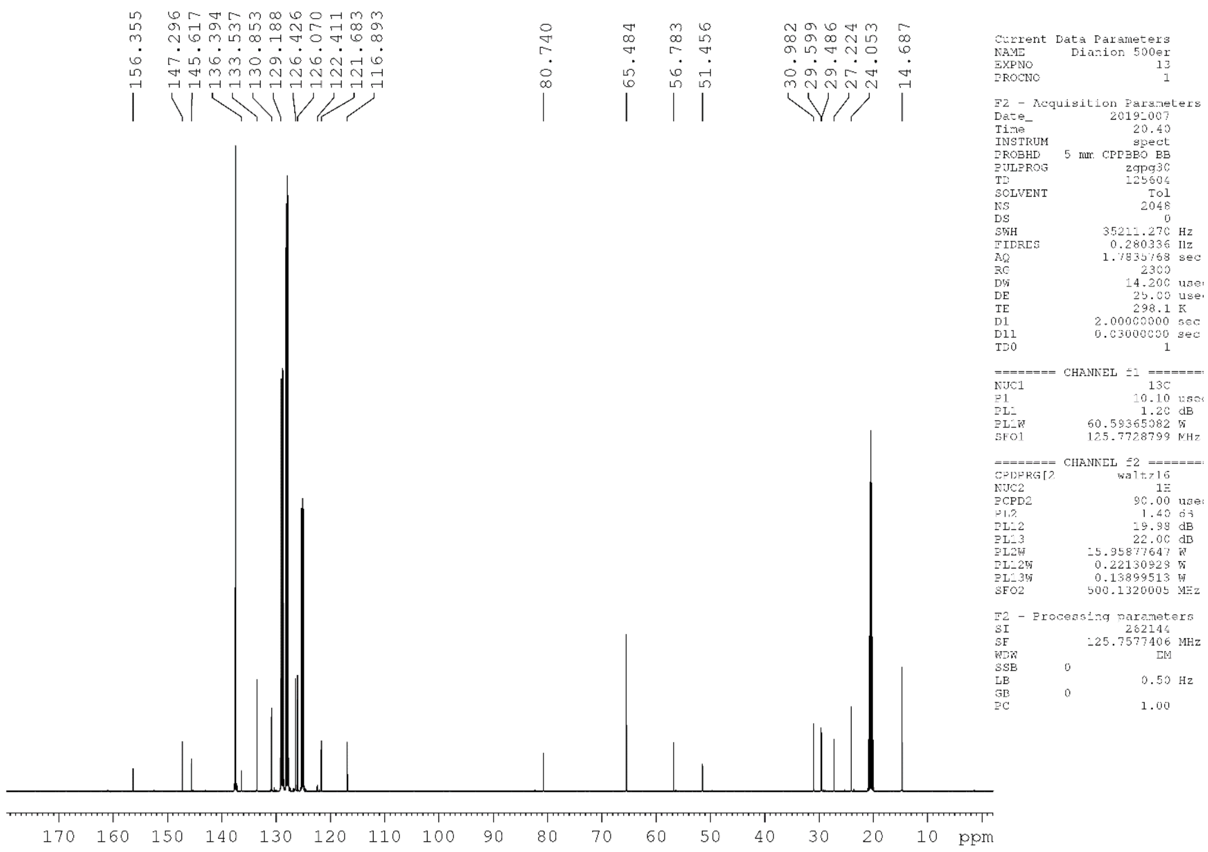

Figure S7. $^{13}C\{^{1}H\}$ NMR spectrum of **2**.

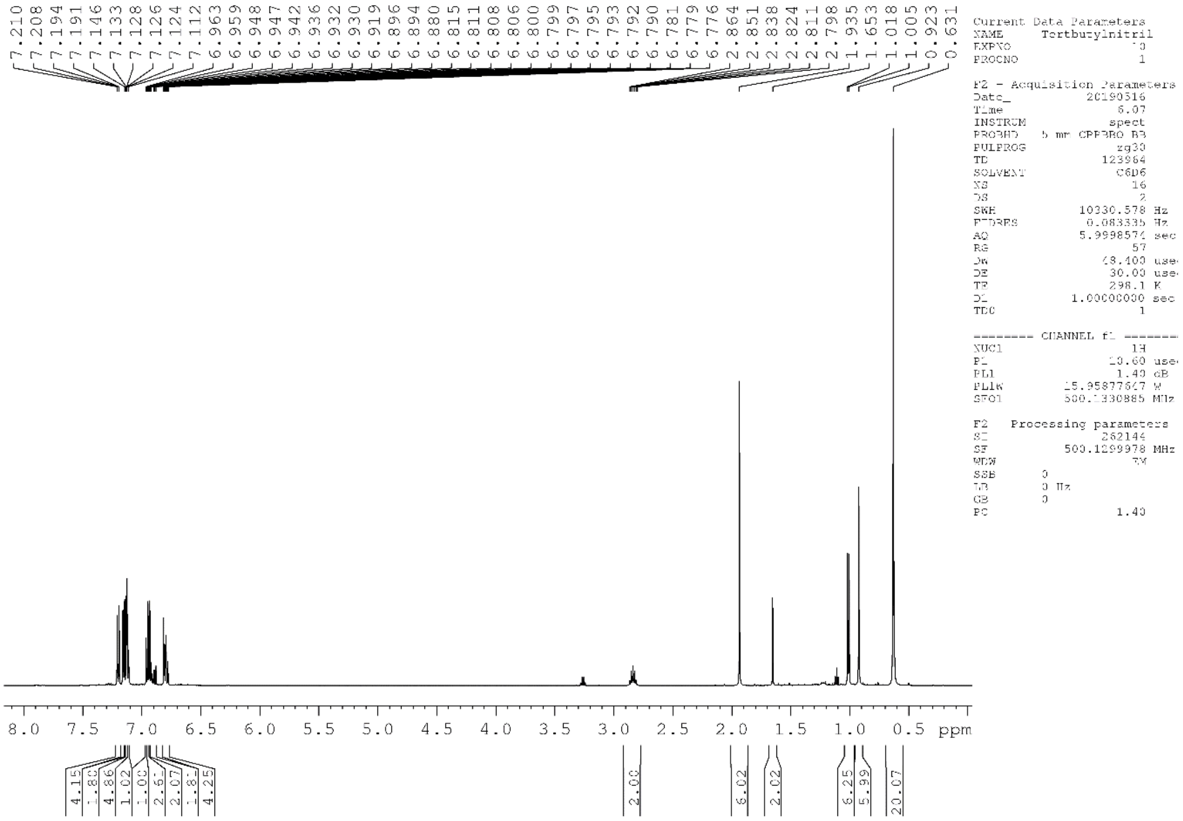

Figure S8. ^{1}H NMR spectrum of **3**.

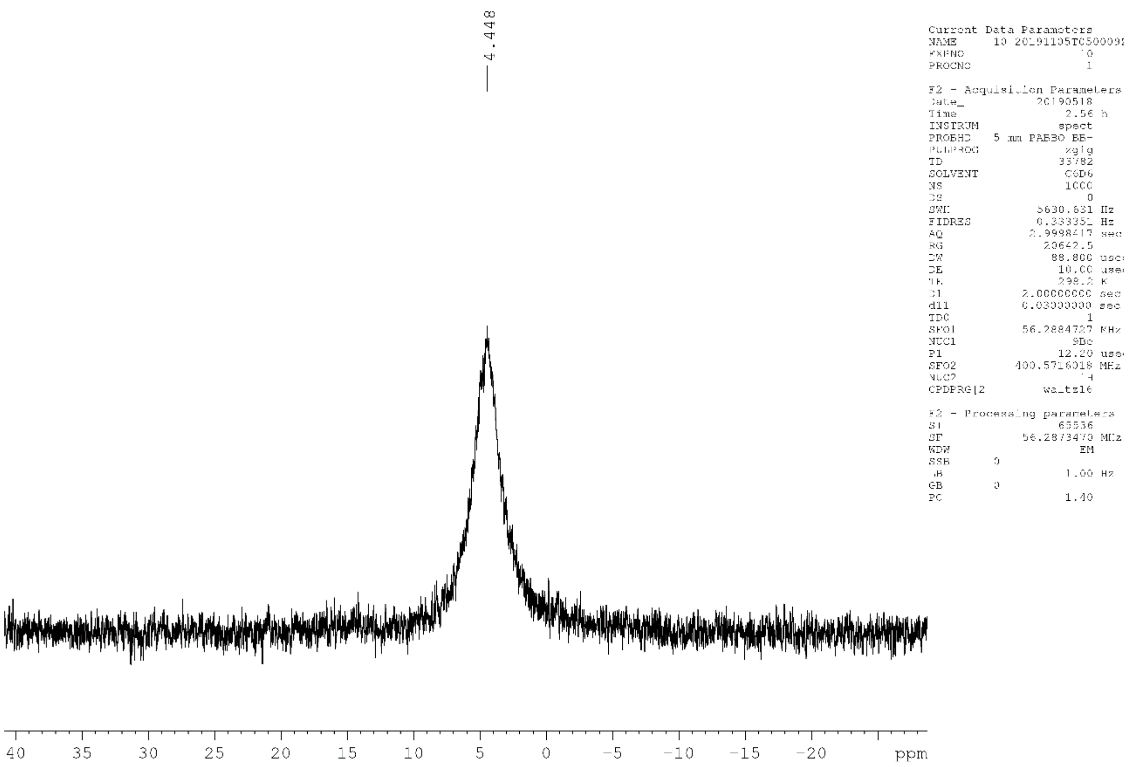

Figure S9. ^{9}Be NMR spectrum of **3**.

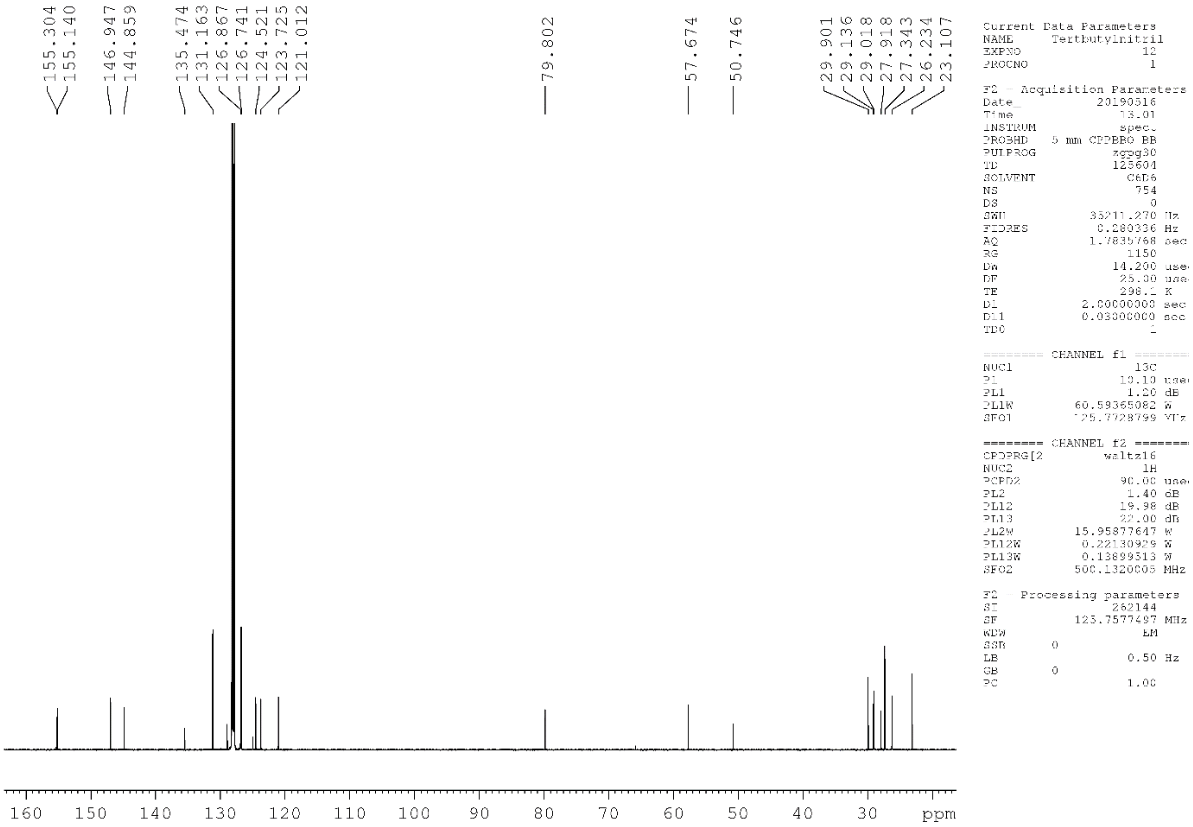

Figure S10. $^{13}C\{^1H\}$ NMR spectrum of **3**.

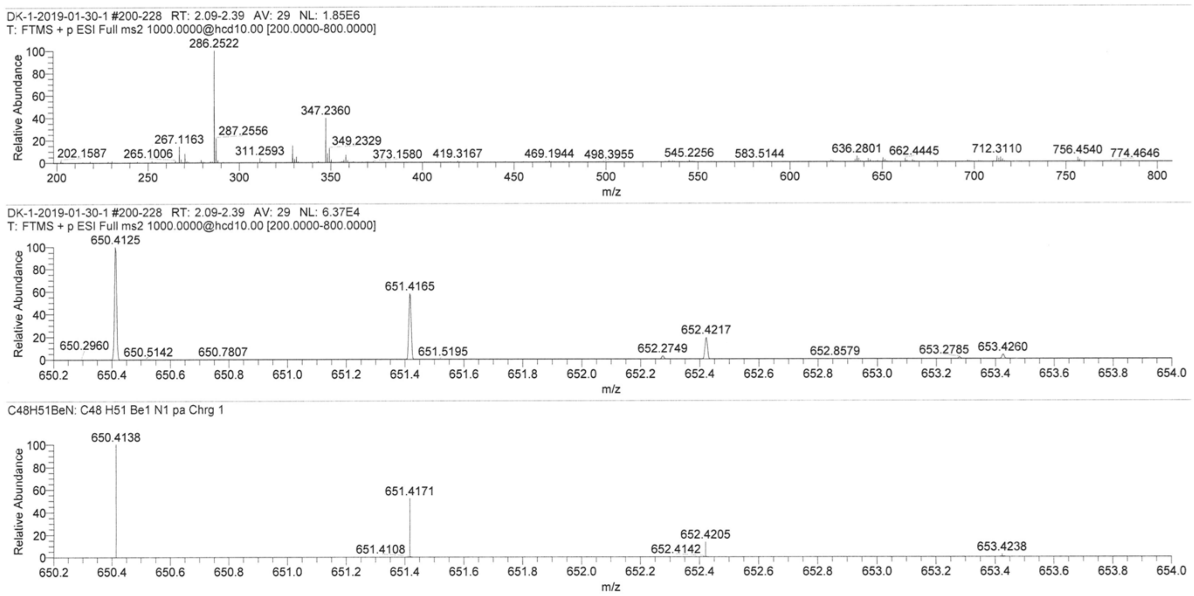

Figure S11. High-resolution LIFDI mass spectrum of **3** (top and middle). Calculated isotope pattern for [M – NC*t*Bu]$^+$ (bottom).

Crystallographic Details

The crystal data of **1** and **3** were collected on a BRUKER X8-APEX II diffractometer with a CCD area detector using Mo-K_{α} radiation and those of **2** on a RIGAKU OXFORD DIFFRACTION SYNERGY-S equipped with a Hybrid Pixel Array Detector HyPix6000 using Cu-K□ radiation. Both diffractometers are equipped with multi-layer mirror monochromators and a FR-591 rotating anode source or a microfocus seal-tube, respectively. The structures were solved using intrinsic phasing methods,[4] refined with the SHELXL program[5] and expanded using Fourier techniques. All non-hydrogen atoms were refined anisotropically. Hydrogen atoms were assigned to idealized geometric positions and included in structure-factor calculations. X-ray data are available free of charge from the Cambridge Crystallographic Data Centre under reference numbers CCDC-1998824 (**1**), -1998823 (**2**), -1998825 (**3**).

Table S1. Crystal data for 1.

Empirical formula	$C_{48}H_{51}BeN$
Formula weight (g·mol^{-1})	650.90
Temperature (K)	100(2)
Radiation, λ (Å)	Mo-K_{α}, 0.71073
Crystal system	Monoclinic
Space group	$P2_1/n$
Unit cell dimensions	
a (Å)	11.000(6)
b (Å)	20.934(11)
c (Å)	16.715(9)
α (°)	90
β (°)	90.968(13)
γ (°)	90
Volume (Å^3)	3848(4)
Z	4
Calculated density (Mg·m^{-3})	1.123
Absorbtion coefficient (mm^{-1})	0.063
F(000)	1400.0
Theta range for collection	3.118 to 52.042°
Reflections collected	35613
Independent reflections	7590
Refinement method	Full-matrix least-squares on F^2
Data / parameters / restraints	7590/0/459
Goodness-of-fit on F^2	1.027
Final R indices [I>2 σ (I)]	R_1 = 0.0426, wR^2 = 0.0998
R indices (all data)	R_1 = 0.0548, wR^2 = 0.1068
Maximum/minimum residual electron density (e·Å^{-3})	0.70 / –0.27

Table S2. Crystal data for 2. Note that one of the ether molecules bound to a lithium cation was disordered. Geometries of all Et_2O residues were constrained using the SAME command. The atomic displacement parameters of atoms belonging to the disordered moiety were restrained with similarity restraint SIMU, rigid-body restraint RIGU and isotropic restraint ISOR.

Empirical formula	$C_{60}H_{81}BeLi_2NO_3$
Formula weight (g·mol^{-1})	887.14
Temperature (K)	100(2)
Radiation, λ (Å)	Cu-K_α, 1.54184
Crystal system	Triclinic
Space group	*P-1*
Unit cell dimensions	
a (Å)	13.0994(4)
b (Å)	13.4502(5)
c (Å)	17.4559(4)
α (°)	79.619(2)
β (°)	84.266(2)
γ (°)	61.279(3)
Volume (Å^3)	2652.68(14)
Z	2
Calculated density (Mg·m^{-3})	1.111
Absorbtion coefficient (mm^{-1})	0.493
F(000)	964.0
Theta range for collection	7.5824 to 154.2938°
Reflections collected	34416
Independent reflections	10764
Refinement method	Full-matrix least-squares on F^2
Data / parameters / restraints	10764/270/664
Goodness-of-fit on F^2	1.060
Final R indices [I>2σ(I)]	R_1 = 0.0566, wR^2 = 0.1587
R indices (all data)	R_1 = 0.0642, wR^2 = 0.1674
Maximum/minimum residual electron density (e·Å^{-3})	0.43 / –0.29

Table S3. Crystal data for 3.

Empirical formula	$C_{53}H_{60}BeN_2$
Formula weight (g·mol^{-1})	734.04
Temperature (K)	100(2)
Radiation, λ (Å)	$Mo_{K\alpha}$ 0.71073
Crystal system	Triclinic
Space group	$P2_1/c$
Unit cell dimensions	
a (Å)	10.0607(7)
b (Å)	17.5293(10)
c (Å)	24.9934(15)
α (°)	90
β (°)	99.159(2)
γ (°)	90
Volume (Å^3)	4351.6(5)
Z	4
Calculated density (Mg·m^{-3})	1.120
Absorbtion coefficient (mm^{-1})	0.063
F(000)	1584
Theta range for collection	2.85 to 54.988°
Reflections collected	27923
Independent reflections	9970
Refinement method	Full-matrix least-squares on F^2
Data / parameters / restraints	9970/28/540
Goodness-of-fit on F^2	1.025
Final R indices [I>2σ(I)]	R_1 = 0.0534, wR^2 = 0.1099
R indices (all data)	R_1 = 0.0925, wR^2 = 0.1263
Maximum/minimum residual electron density (e·Å^{-3})	0.43 / –0.28

Computational Details

Geometry optimizations at the B3LYP-D3(BJ)/def2-SVP[6-12] level of theory were performed for **1**, **2**, **3** and for a model compound where the Li(OEt_2) moieties of **2** were removed, thus leading to the naked dianion [(CAAC)BeC_4Ph_4]$^{2-}$. Stability tests were then conducted on the single-determinant restricted closed-shell wavefunctions of the optimized geometries at singlet multiplicities, following a previously described protocol.[13,14] While no instability was observed for the closed-shell wavefunction of **1**, **2** and **3**, a restricted Hartree-Fock (RHF)/unrestricted Hartree-Fock (UHF) instability was found for [(CAAC)BeC_4Ph_4]$^{2-}$. After a stable open-shell singlet wavefunction was generated for [(CAAC)BeC_4Ph_4]$^{2-}$ at the UB3LYP-D3(BJ)/def2-SVP level of theory, a new geometry optimization was performed for the system, and atomic spin density values were obtained at the optimized structure by calculating the Mulliken population analysis. For obtaining the adiabatic singlet-triplet energy gap of [(CAAC)BeC_4Ph_4]$^{2-}$, the geometry of the triplet state was also optimized. All systems were characterized as minimum energy structures by vibrational frequency calculations, which indicated that all Hessian eigenvalues were positive. In order to confirm the open-shell character of the singlet naked beryllole dianion [(CAAC)BeC_4Ph_4]$^{2-}$, single-point calculations were performed for the full system using high-level complete active space self-consistent field (CASSCF)[15] and N-electron valence state second-order perturbation theory (NEVPT2)[16-18] calculations with def2-SVP and the minimally-augmented minimally augmented diffuse (ma-def2-SVP) basis sets.[19] Given the large size of the system, the CASSCF calculations were performed using the resolution-of-the-identity approximation for Coulomb integrals (RI-J)[20,21] in combination with the numerical chain-of-sphere integration for the HF exchange integrals (COSX).[22,23] In turn, the NEVPT2 calculations were carried out using the DLPNO-NEVPT2 approximation.[24] The biradical character y,[25-27] which can vary from 0 (closed-shell system) to 1 (pure biradical state), was obtained for [(CAAC)BeC_4Ph_4]$^{2-}$ using the natural orbital occupancy numbers (NOON)[28] of a CASSCF(4,3) calculation.[29] Additionally, NEVPT2 calculations were performed, using CASSCF(4,3) and CASSCF(6,6) wavefunctions as reference, for both the singlet and triplet multiplicities for an accurate determination of the adiabatic singlet-triplet gap of [(CAAC)BeC_4Ph_4]$^{2-}$, herein calculated as $\Delta_{T-S} = E_{triplet} - E_{singlet}$. The vertical singlet-triplet gap of **2** was also obtained for comparison. The y index[25-27] was calculated using the weight of the doubly-excited configuration in a CASSCF(4,3) approach, and is given by the following expression:

$$y = 1 - \frac{2T}{1 + T^2} \qquad (S1)$$

where T is the orbital overlap of the highest occupied natural orbital (HONO) and the lowest unoccupied natural orbital (LUNO), and is obtained by collecting the occupation numbers (ON) of the proper natural orbitals:

$$T = \frac{ON_{HONO} - ON_{LUNO}}{2} \qquad (S2)$$

The nature of the Be–CAAC bond was investigated using the energy decomposition analysis with natural orbitals for chemical valence (EDA-NOCV) method.[30,31] These calculations were performed using a truncated model of **1** where the phenyl substituents of the BeC_4 ring, the methyl groups at CAAC and the diisopropyl groups at the Dip group were replaced by hydrogen atoms. The chosen level of theory was B3LYP-D3(BJ)/TZ2P. Two distinct scenarios were tested, depending on the choice of the fragments. In the first scenario, the BeC_4H_4 and the CAAC fragments were calculated in their closed-shell singlet states, and their interaction gives rise to a donor-acceptor bonding scheme as a result of σ-donation of the CAAC to the beryllium atom. In the second scenario, the BeC_4H_4 fragment was calculated with an additional negative charge, whereas the CAAC bore a positive charge. The bond situation in this case would then be an electron-sharing bond between Be and CAAC. The more appropriate choice of fragments is the one in which the orbital interaction energy has the value closest to zero.[31]

In order to describe the beryllium systems obtained herein in terms of their (non/anti)aromaticity, nucleus-independent chemical shift (NICS) calculations[32,33] were performed through the gauge-independent atomic orbital (GIAO) method.[34-36] These quantities were calculated by placing ghost atoms at the ring centroid and at several distances along the axis perpendicular to the ring plane, with a step size of 0.1 Å. For each one of these points, the zz-component of the magnetic shielding tensor was obtained, and the plot of these values with respect to the distance to the ring centroid gives the $NICS_{zz}$-scan curve.[37,38] These curves were obtained for the experimentally-realized compounds **1-3**, along with a number of other relevant molecules for comparison, from single-point calculations at the B3LYP/6-311++G** level of structures optimized at the B3LYP/6-31+G* level. The $NICS_{zz}$-scan plot of the dilithiated beryllole dianion $[Li(OEt_2)]_2[(CAAC)BeC_4Ph_4]$ (**2**) was obtained by subtracting the contribution of the Li^+ ions in their equilibrium positions. The aromatic stabilization energies (ASEs)[39,40] obtained herein were calculated at the B3LYP/6-311++G** level of theory from structures optimized at the B3LYP/6-31+G* level. The zero-point-energy-corrected electronic energies were used for estimating the ASEs. Calculations using the CBS-QB3 method[41,42] of selected systems were used to validate the DFT protocol. All DFT and NICS calculations were performed with the Gaussian 16, Revision B.01 software.[43] CASSCF and NEVPT2 calculations were performed with the Orca 4.1.1 software.[44] The EDA-NOCV calculations

were performed with ADF 2019.[45] Pictures of molecular structures, orbitals and densities were visualized and generated with Chemcraft,[46] ADFView and Gaussview.

The anisotropy of the induced current density (ACID) method[47,48] was applied to assess the antiaromaticity of CAAC-stabilized BeC_4 rings in comparison to $C_5H_5^+$ and C_4H_4BH. NMR properties were calculated using the continuous set of gauge transformations (CSGT) method.[36,49,50] ACID plots were obtained using the ACID software package.

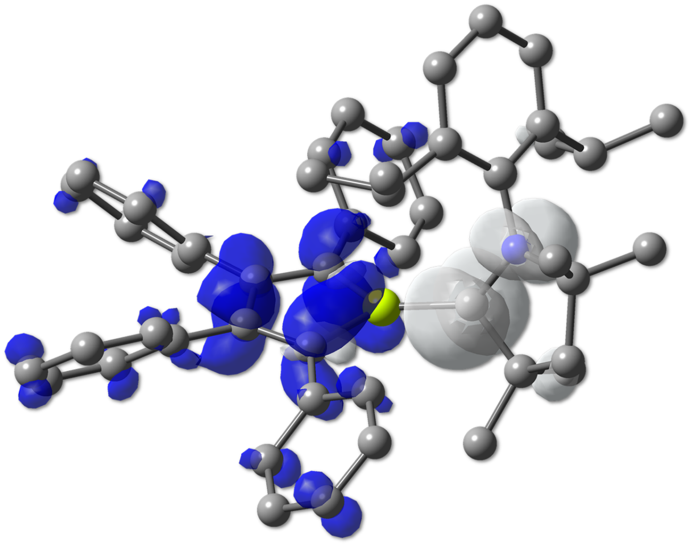

Figure S12. Spin density of the naked singlet beryllole dianion $[(CAAC)BeC_4Ph_4]^{2-}$ at the B3LYP-D3(BJ)/def2-SVP level of theory. Blue depicts the alpha spin density, while white represents the beta spin density. Contour value: 0.002.

Table S4. **Dependence of the tilt angle of the CAAC unit out of the BeC_4 plane with respect to the R_1-R_3 substituents.**

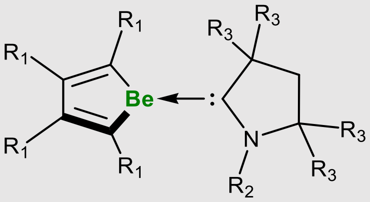

R_1	R_2	R_3	Tilt Angle	Optimized Structure
Ph	Dip	Me	148.0	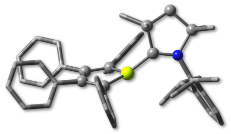
Me	Dip	Me	160.9	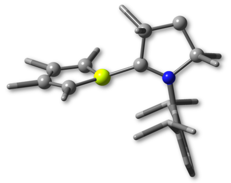
H	Dip	Me	163.1	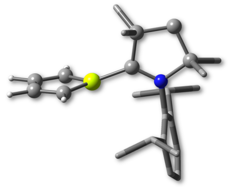
H	Ph	Me	173.4	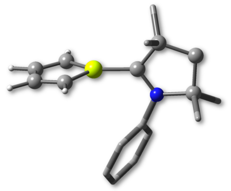
H	Me	Me	170.0	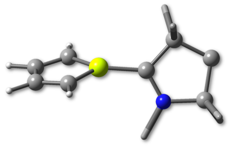
H	H	H	179.8	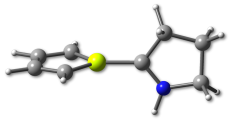

Table S5. **CASSCF/NEVPT2 results of the naked singlet beryllole dianion $[(CAAC)BeC_4Ph_4]^{2-}$ using ma-def2-SVP basis set.** The active space is composed of four electrons and three orbitals.

**CASSCF(4,3)/ma-def2-SVP**	
Electronic energy (singlet), E_h	–1914.887423
Electronic energy (triplet), E_h	–1914.885840
Weight of the 2 2 0 configuration (singlet)	0.67573
Weight of the 2 0 2 configuration (singlet)	0.32424
Occupation of the HONO (ON^{HONO}, singlet)	1.3515
Occupation of the LUNO (ON^{LUNO}, singlet)	0.6485
HONO-LUNO orbital overlap (T, singlet)	0.3515
Biradical character (y, singlet)	0.3743 (37.4%)
**NEVPT2/CASSCF(4,3)/ma-def2-SVP**	
Electronic energy (singlet), E_h	–1921.896582
Electronic energy (triplet), E_h	–1921.891299
Singlet-triplet gap ($\Delta_{T-S} = E_{triplet} - E_{singlet}$, kcal mol^{-1})	3.3

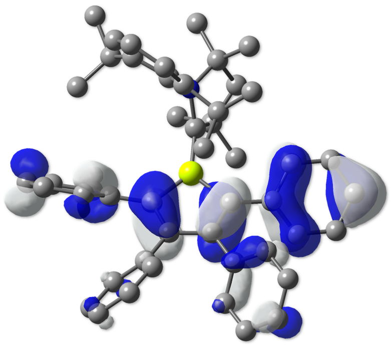

HONO-1
ON: 2.0000

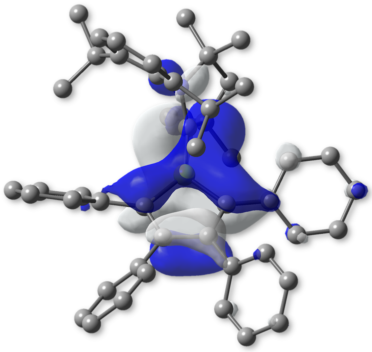

HONO
ON: 1.3515

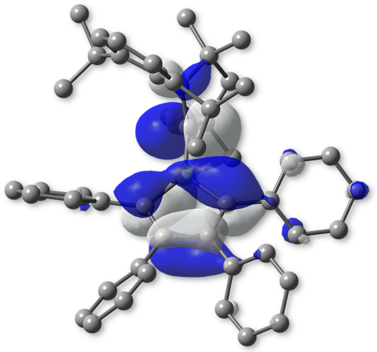

LUNO
ON: 0.6485

Figure S13. Active space CASSCF(4,3)/ma-def2-SVP natural orbitals of $[(CAAC)BeC_4Ph_4]^{2-}$. Contour value: 0.03.

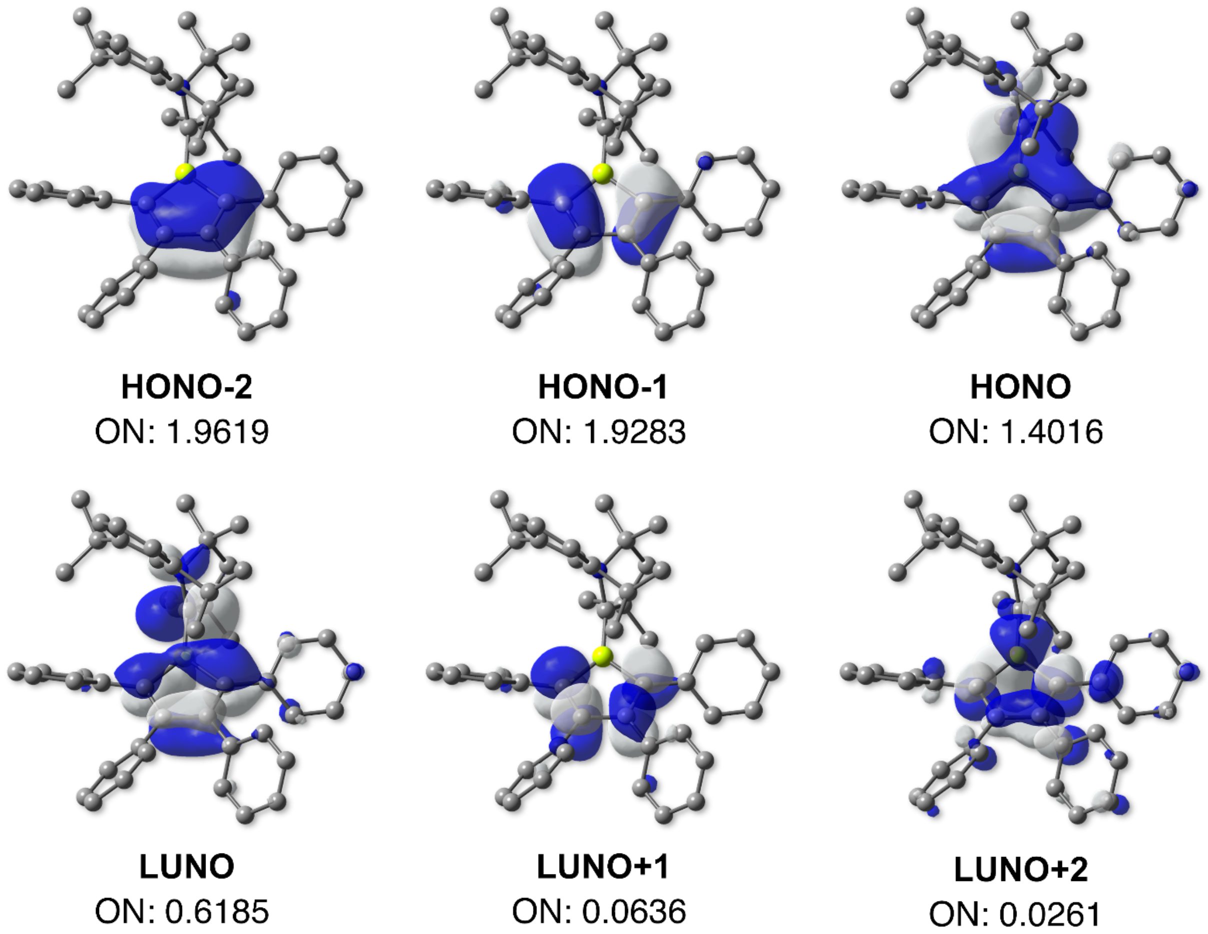

Figure S14. Active space CASSCF(6,6)/def2-SVP natural orbitals of $[(CAAC)BeC_4Ph_4]^{2-}$. Contour value: 0.03.

Table S6. **Energy terms involved in the calculation of the aromatic stabilization energies (ASEs).**

**Electronic Energy, E_h**					
	X = CH^+	X = BH	X = Be(CAAC)	X = BeH^-	X = Be(CAAC)(*t*BuCN)
C_4H_4X	–193.2071	–180.2667	–1005.3398	–170.2042	–1255.1176
C_4H_6X	–194.4889	–181.5215	–1006.5742	–171.4355	–1256.3157
C_4H_8X	–195.6864	–182.7417	–1007.7965	–172.6582	–1257.5133
**Zero-point Energy (ZPE), E_h**					
	X = CH^+	X = BH	X = Be(CAAC)	X = BeH^-	X = Be(CAAC)(*t*BuCN)
C_4H_4X	0.0781	0.0769	0.5364	0.0698	0.6687
C_4H_6X	0.1044	0.1002	0.5589	0.0919	0.6912
C_4H_8X	0.1260	0.1228	0.5817	0.1143	0.7146
**E+ZPE, E_h**					
	X = CH^+	X = BH	X = Be(CAAC)	X = BeH^-	X = Be(CAAC)(*t*BuCN)
C_4H_4X	–193.1289	–180.1899	–1004.8034	–170.1344	–1254.4489
C_4H_6X	–194.3845	–181.4213	–1006.0153	–171.3436	–1255.6245
C_4H_8X	–195.5605	–182.6189	–1007.2148	–172.5439	–1256.7987
**Aromatic stabilization energy, kcal mol^{-1}**					
	X = CH^+	X = BH	X = Be(CAAC)	X = BeH^-	X = Be(CAAC)(*t*BuCN)
ASE	–49.9	–21.2	–7.8	–5.6	–0.9

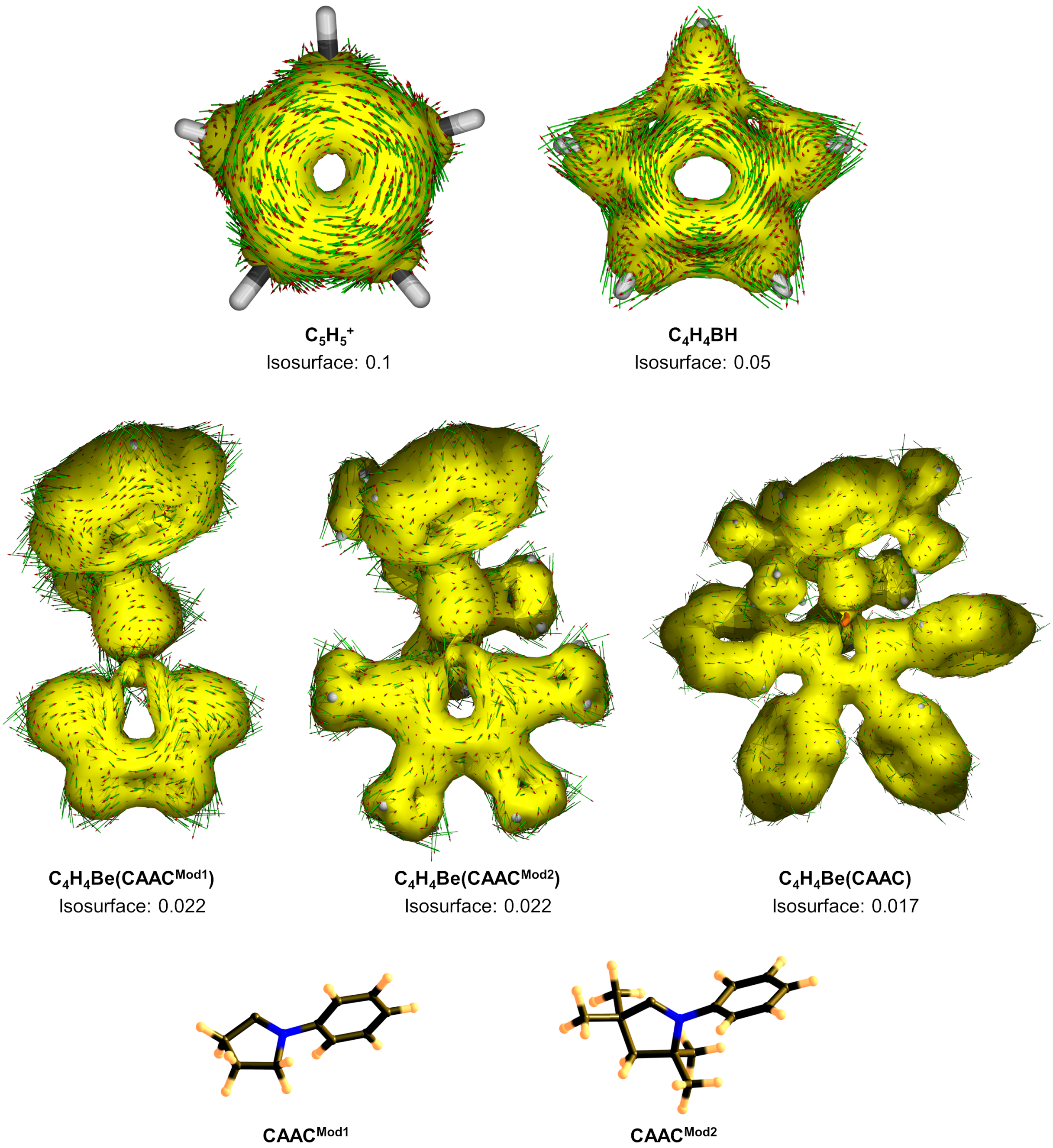

Figure S15. ACID plots (B3LYP/6-311++G**) of $C_5H_5^+$, C_4H_4BH and distinct CAAC-stabilized berylloles.

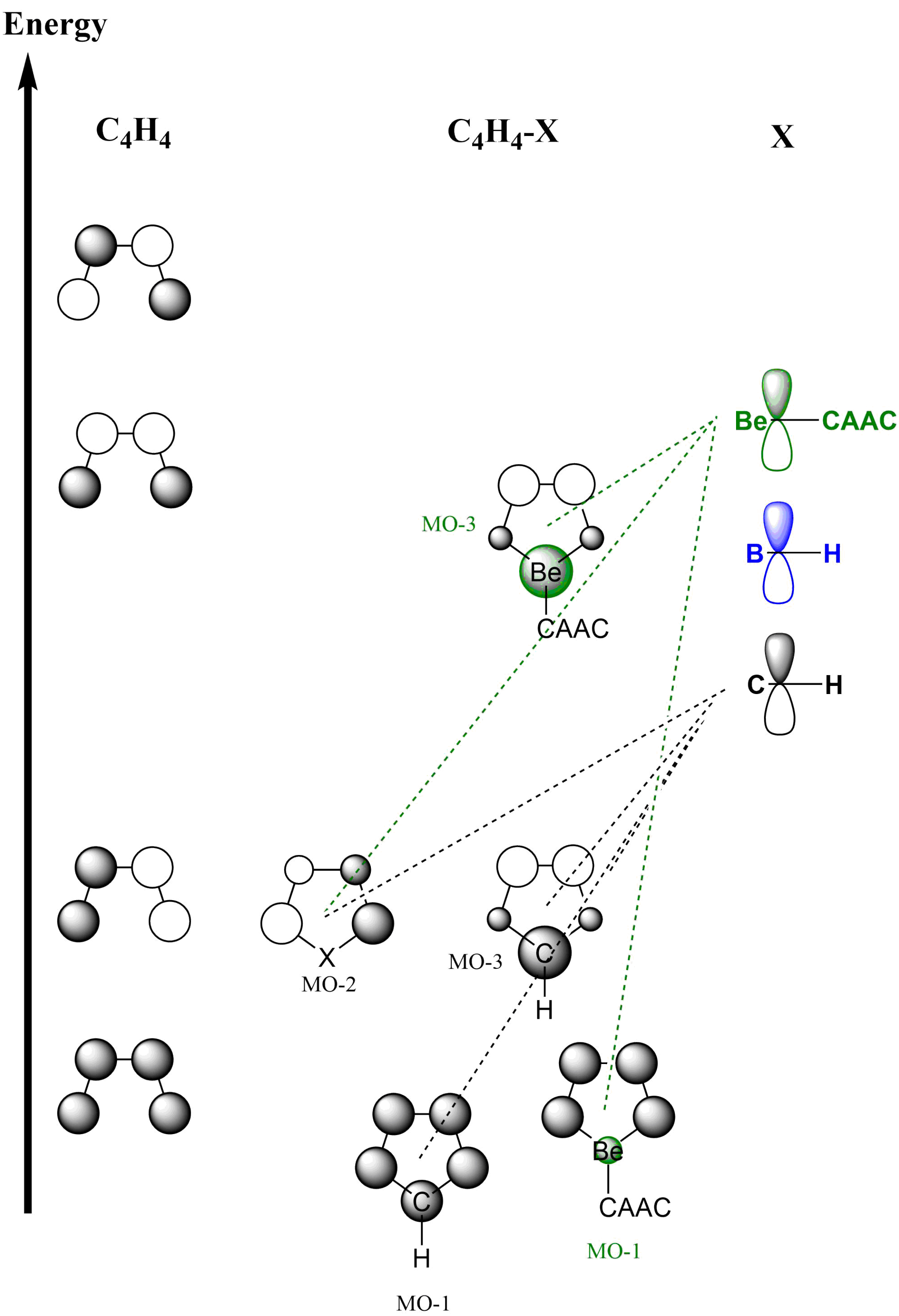

Figure S16. Sketch of the variation of the MOs for $[XC_4H_4]$ (X = $[HC]^+$ (black), [HB] (blue), and [(CAAC)Be] (green). The black ($[HC]^+$) and green ([(CAAC)Be]) dotted lines indicate the correlation between the X-fragment orbital and the corresponding X-C_4H_4 orbitals. Carbon MOs are given in black while Be contributions are given in green. Because the fragment orbital energies of X increase going from X = $[HC]^+$ to [HB] and [(CAAC)Be], the shapes and energies of MO-1 and MO-3 change. For more information see the main text. The situation for X = [HB] (not shown) lies in between the other two. MO-2 remains nearly unchanged because X does not contribute. The computed shapes of the orbitals are given in Figure 3 of the main text.

Table S7. **EDA-NOCV results for the Be–CAAC interaction considering donor-acceptor and electron-sharing bonding schemes.**

Energy Terms	Donor-Acceptor BeC_4H_4 (singlet) + CAAC (singlet)	Donor-Acceptor $BeC_4H_4^-$ (doublet) + $CAAC^+$ (doublet)
ΔE_{int}	–62.7	–221.3
ΔE_{Pauli}	80.0	222.5
ΔE_{elstat}[a]	–85.0 (59.6%)	–222.5 (50.1%)
ΔE_{disp}[a]	–5.7 (4.0%)	–5.7 (1.3%)
ΔE_{orb}[a]	–51.9 (36.4%)	–215.6 (48.6%)
$\Delta E_{orb(1)}$[b]	–33.7 (64.8%)	–191.6 (88.9%)
$\Delta E_{orb(2)}$[b]	–9.5 (18.3%)	–14.3 (6.6%)
$\Delta E_{orb(rest)}$[b]	–8.7 (16.8%)	–9.7 (4.5%)

[a] The values in parentheses show the weight of each contribution with respect to the total attractive interaction. [b] The values in parentheses show the weight of each contribution with respect to the total orbital interaction, ΔE_{orb}.

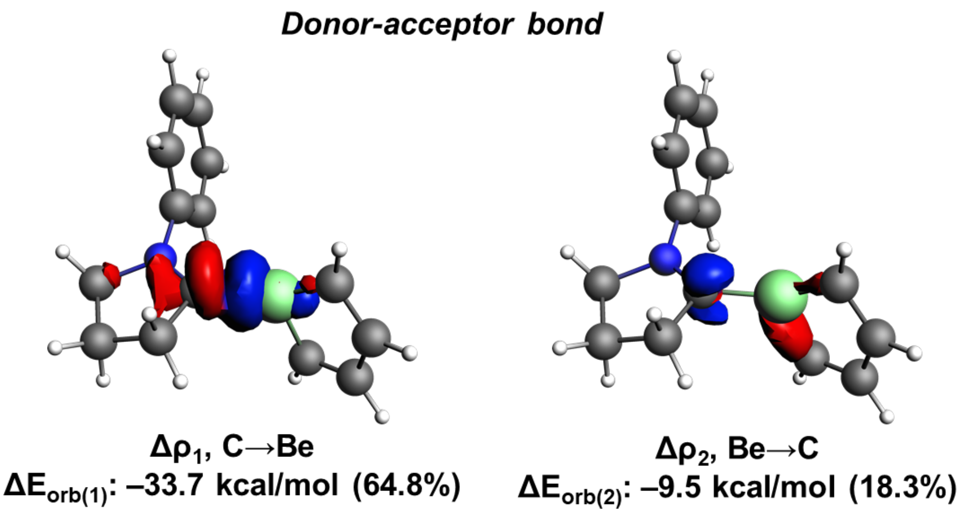

Figure S17. Deformation density plots of the two main bonding configurations that contribute to the total orbital interactions in the EDA-NOCV description of the truncated model of **1** from BeC_4H_4 (singlet) and CAAC (singlet) fragments. Isovalues: 0.0035 au. Charge flows from red to blue.

Cartesian Coordinates

**Compound 1, B3LYP-D3(BJ)/def2-SVP**			
**Lowest frequency: 15.91 cm^{-1}**			
**E+ZPE = –1927.331264 E$_h$**			
Be	-0.059619000	0.129399000	-0.100208000
C	0.991489000	-1.297528000	-0.169587000
C	2.242147000	-0.744011000	-0.207719000
C	2.292163000	0.766182000	-0.312341000
C	1.085994000	1.399625000	-0.434248000
C	-1.437127000	0.055555000	0.956204000
N	-2.707145000	-0.202067000	0.760702000
C	-3.478041000	-0.671164000	1.996082000
C	-2.355236000	-0.678638000	3.056970000
H	-2.698222000	-0.257566000	4.012753000
H	-2.034944000	-1.712620000	3.252308000
C	-1.175385000	0.118253000	2.457684000
C	-4.024230000	-2.076175000	1.734437000
H	-4.781331000	-2.069431000	0.937716000
H	-4.499947000	-2.450692000	2.653174000
H	-3.224843000	-2.773982000	1.453766000
C	-4.644797000	0.252065000	2.357653000
H	-5.337098000	0.370474000	1.512673000
H	-4.314410000	1.244992000	2.684977000
H	-5.203429000	-0.203243000	3.188570000
C	0.196134000	-0.402136000	2.904607000
H	0.341337000	-1.461382000	2.662315000
H	0.293742000	-0.278749000	3.994566000
H	1.005157000	0.164908000	2.422007000
C	-1.245864000	1.624559000	2.818551000
H	-2.238056000	2.054574000	2.631336000
H	-0.514600000	2.204056000	2.237196000
H	-1.018373000	1.748344000	3.888534000
C	-3.399289000	-0.032519000	-0.507267000
C	-4.018869000	1.218136000	-0.754074000
C	-4.790627000	1.355547000	-1.913443000
H	-5.274546000	2.310930000	-2.120529000

C	-4.937116000	0.302160000	-2.813027000
H	-5.552330000	0.426011000	-3.707349000
C	-4.267732000	-0.894252000	-2.585077000
H	-4.342463000	-1.701598000	-3.315665000
C	-3.475021000	-1.086115000	-1.443111000
C	-3.779335000	2.439659000	0.120480000
H	-3.420876000	2.092945000	1.092168000
C	-2.648280000	3.282745000	-0.482623000
H	-2.926082000	3.670193000	-1.475461000
H	-1.734810000	2.688306000	-0.602934000
H	-2.400480000	4.140659000	0.161848000
C	-5.036846000	3.278001000	0.374377000
H	-5.391811000	3.775030000	-0.541740000
H	-4.816925000	4.072078000	1.105059000
H	-5.864561000	2.668802000	0.769257000
C	-2.659075000	-2.361422000	-1.339587000
H	-2.137554000	-2.361197000	-0.378032000
C	-1.576920000	-2.373116000	-2.431959000
H	-2.028647000	-2.353607000	-3.436430000
H	-0.960464000	-3.277812000	-2.345122000
H	-0.905859000	-1.506341000	-2.340450000
C	-3.504652000	-3.639656000	-1.413947000
H	-3.957234000	-3.768741000	-2.410059000
H	-4.319195000	-3.642553000	-0.675026000
H	-2.864948000	-4.514905000	-1.223269000
C	0.751607000	-2.746714000	-0.148426000
C	1.415480000	-3.627443000	-1.033273000
H	2.147283000	-3.217789000	-1.730397000
C	1.140053000	-4.993290000	-1.046900000
H	1.668101000	-5.641290000	-1.751511000
C	0.193825000	-5.539277000	-0.171108000
H	-0.020147000	-6.610573000	-0.182197000
C	-0.473750000	-4.690859000	0.715817000
H	-1.216657000	-5.096130000	1.408614000
C	-0.203238000	-3.321122000	0.716423000
H	-0.744447000	-2.674072000	1.406636000
C	3.511937000	-1.517485000	-0.137313000

C	4.521813000	-1.345594000	-1.100243000
H	4.374817000	-0.622975000	-1.904910000
C	5.702967000	-2.086196000	-1.039191000
H	6.472992000	-1.941003000	-1.801135000
C	5.905821000	-3.004424000	-0.004622000
H	6.833614000	-3.579602000	0.046903000
C	4.914974000	-3.176814000	0.966531000
H	5.065426000	-3.888843000	1.782104000
C	3.730885000	-2.441961000	0.898293000
H	2.956012000	-2.580378000	1.654532000
C	3.591441000	1.485046000	-0.207148000
C	3.966988000	2.442409000	-1.165187000
H	3.304260000	2.634945000	-2.009883000
C	5.169168000	3.141742000	-1.048043000
H	5.442111000	3.879311000	-1.807125000
C	6.020493000	2.902707000	0.034377000
H	6.961654000	3.450422000	0.126944000
C	5.659330000	1.954608000	0.996261000
H	6.317744000	1.758154000	1.846328000
C	4.461452000	1.250109000	0.872931000
H	4.192861000	0.504784000	1.623639000
C	0.962455000	2.868087000	-0.465051000
C	0.476391000	3.527452000	-1.610432000
H	0.217341000	2.930107000	-2.488486000
C	0.323385000	4.914388000	-1.639016000
H	-0.048003000	5.399660000	-2.545729000
C	0.635068000	5.682669000	-0.512784000
H	0.508835000	6.767892000	-0.531344000
C	1.117309000	5.045958000	0.634792000
H	1.375910000	5.634953000	1.518984000
C	1.285946000	3.660075000	0.654520000
H	1.690869000	3.172477000	1.544344000

Compound 2, B3LYP-D3(BJ)/def2-SVP

Lowest frequency: 19.36 cm^{-1}

E+ZPE = –2409.291868 E$_h$

Li	0.308952000	-0.342065000	1.357656000
Li	1.659580000	0.641667000	-1.660173000
Be	-0.427219000	0.150631000	-0.755595000
C	0.527535000	1.379532000	0.020428000
C	1.806156000	0.769596000	0.416927000
C	1.915452000	-0.609967000	0.007801000
C	0.763041000	-1.083162000	-0.756513000
C	-2.074631000	0.450271000	-1.311621000
N	-3.214805000	0.422085000	-0.644313000
C	-4.413260000	1.057205000	-1.357269000
C	-3.710212000	1.717173000	-2.556641000
H	-3.529326000	2.779982000	-2.336687000
H	-4.324230000	1.666121000	-3.467488000
C	-2.356526000	0.997801000	-2.709149000
C	-5.450555000	0.002772000	-1.757085000
H	-6.290553000	0.505846000	-2.258039000
H	-5.847000000	-0.510494000	-0.870415000
H	-5.052186000	-0.749725000	-2.445575000
C	-5.114070000	2.093634000	-0.480222000
H	-5.937380000	2.537292000	-1.060002000
H	-4.440785000	2.906136000	-0.181728000
H	-5.546065000	1.636510000	0.419982000
C	-2.457775000	-0.225018000	-3.651988000
H	-2.604951000	0.129085000	-4.684331000
H	-3.293689000	-0.889960000	-3.400868000
H	-1.538394000	-0.822146000	-3.612950000
C	-1.253410000	1.914838000	-3.245007000
H	-1.546096000	2.307242000	-4.232285000
H	-0.315657000	1.354484000	-3.365864000
H	-1.059667000	2.760089000	-2.573149000
C	-3.416426000	-0.289004000	0.611896000
C	-3.413548000	0.391796000	1.854920000
C	-3.692055000	-0.344870000	3.015363000
H	-3.687336000	0.170241000	3.977378000

C	-3.981663000	-1.703358000	2.968749000
H	-4.208081000	-2.251303000	3.886634000
C	-3.967624000	-2.360156000	1.743667000
H	-4.170802000	-3.431570000	1.705321000
C	-3.679609000	-1.682184000	0.551870000
C	-3.090835000	1.867347000	2.035276000
H	-3.006168000	2.317170000	1.045276000
C	-1.731607000	2.057320000	2.720291000
H	-1.494031000	3.127848000	2.805637000
H	-0.933515000	1.596876000	2.120889000
H	-1.733364000	1.619183000	3.730439000
C	-4.182461000	2.626030000	2.805932000
H	-3.972649000	3.706849000	2.781618000
H	-4.214352000	2.324639000	3.864808000
H	-5.184758000	2.462784000	2.384443000
C	-3.594837000	-2.514871000	-0.719791000
H	-3.447897000	-1.837497000	-1.566071000
C	-4.860661000	-3.342005000	-0.987550000
H	-4.780196000	-3.843576000	-1.964898000
H	-5.772721000	-2.728084000	-0.992310000
H	-4.993417000	-4.130602000	-0.230460000
C	-2.358456000	-3.418193000	-0.657964000
H	-2.205537000	-3.953746000	-1.606423000
H	-2.450999000	-4.167130000	0.144730000
H	-1.460621000	-2.821227000	-0.472206000
C	0.339802000	2.833572000	-0.036913000
C	-0.961364000	3.381867000	-0.189185000
H	-1.793827000	2.685879000	-0.237381000
C	-1.214397000	4.745640000	-0.300823000
H	-2.246868000	5.093210000	-0.411367000
C	-0.161098000	5.668379000	-0.280135000
H	-0.348916000	6.741222000	-0.363208000
C	1.140346000	5.169465000	-0.166672000
H	1.989644000	5.859481000	-0.172464000
C	1.387147000	3.800332000	-0.054515000
H	2.422358000	3.471000000	0.003250000
C	2.871461000	1.444341000	1.199969000

C	2.548158000	2.229822000	2.324903000
H	1.497147000	2.357801000	2.586212000
C	3.532472000	2.864740000	3.081046000
H	3.243913000	3.467347000	3.946667000
C	4.881849000	2.738239000	2.733956000
H	5.655548000	3.234170000	3.325182000
C	5.224539000	1.977525000	1.611795000
H	6.272995000	1.876002000	1.318649000
C	4.234632000	1.347767000	0.855930000
H	4.518139000	0.766285000	-0.021746000
C	3.063458000	-1.470638000	0.395581000
C	3.475723000	-1.573364000	1.739642000
H	2.948427000	-0.994349000	2.498095000
C	4.541263000	-2.391790000	2.116527000
H	4.837681000	-2.441994000	3.167803000
C	5.226229000	-3.145727000	1.158415000
H	6.059921000	-3.788745000	1.450927000
C	4.824677000	-3.069053000	-0.178618000
H	5.342542000	-3.657681000	-0.940629000
C	3.759847000	-2.247539000	-0.550310000
H	3.447871000	-2.216518000	-1.593399000
C	0.749919000	-2.432736000	-1.346905000
C	1.042776000	-3.623703000	-0.638459000
H	1.339392000	-3.555521000	0.405943000
C	0.960185000	-4.881020000	-1.234635000
H	1.189188000	-5.771353000	-0.641595000
C	0.588988000	-5.014224000	-2.577773000
H	0.523505000	-6.000467000	-3.043525000
C	0.318342000	-3.856128000	-3.313436000
H	0.043921000	-3.929795000	-4.370041000
C	0.406240000	-2.600427000	-2.709362000
H	0.216008000	-1.701172000	-3.299187000
O	-0.007787000	-1.383738000	2.917656000
C	-0.418208000	-2.719472000	2.590978000
H	-0.566099000	-2.720006000	1.502983000
H	-1.399965000	-2.915782000	3.053712000
H	0.733139000	-3.838155000	4.079949000

C	-0.131687000	-1.033352000	4.294817000
O	2.712306000	1.027418000	-3.189318000
C	3.477496000	-0.070402000	-3.694600000
H	3.509770000	0.003295000	-4.797925000
H	2.894299000	-0.968609000	-3.439664000
C	4.879729000	-0.150760000	-3.114661000
H	5.377478000	-1.064326000	-3.474970000
H	4.846049000	-0.200801000	-2.016923000
H	5.498414000	0.709346000	-3.412523000
C	3.202044000	2.332358000	-3.516189000
H	3.680611000	2.286860000	-4.511413000
H	3.975239000	2.627352000	-2.784484000
C	2.055363000	3.321541000	-3.517511000
H	2.436776000	4.332391000	-3.728830000
H	1.555915000	3.359308000	-2.538422000
H	1.313371000	3.059595000	-4.286054000
C	0.615312000	-3.759614000	2.988122000
H	0.305529000	-4.747699000	2.613628000
H	1.595471000	-3.514927000	2.552717000
C	0.790293000	0.122236000	4.618542000
H	0.688982000	0.398233000	5.679632000
H	1.840226000	-0.141467000	4.424873000
H	0.549735000	1.006341000	4.014943000
H	0.125932000	-1.907193000	4.916122000
H	-1.187258000	-0.777667000	4.501422000

Compound 3, B3LYP-D3(BJ)/def2-SVP

Lowest frequency: 5.64 cm^{-1}

E+ZPE = –2177.773735 E$_h$

N	-2.400652000	0.178463000	-1.488656000
N	-0.807058000	-0.392873000	1.472720000
C	5.678664000	3.572989000	0.228845000
H	6.532450000	4.253132000	0.283610000
C	5.540621000	2.537936000	1.158741000
H	6.288472000	2.405266000	1.945015000
C	4.453620000	1.666232000	1.084076000

H	4.358719000	0.853921000	1.807103000
C	3.473346000	1.813641000	0.086632000
C	2.299801000	0.897087000	0.020761000
C	1.000587000	1.322255000	-0.006396000
C	-1.106914000	-0.077465000	-1.445634000
C	-3.314627000	-0.007377000	-0.374326000
C	-3.724263000	1.080802000	0.429985000
C	-4.710839000	0.850215000	1.400509000
H	-5.045837000	1.685462000	2.018465000
C	-5.255330000	-0.412949000	1.601853000
H	-6.026314000	-0.565936000	2.360958000
C	-2.290558000	-3.292074000	0.013254000
H	-1.454263000	-2.641577000	0.289045000
H	-1.867507000	-4.159601000	-0.515668000
H	-2.765698000	-3.655285000	0.939738000
C	-3.297155000	-2.544106000	-0.870292000
H	-2.741803000	-2.208049000	-1.748272000
C	-3.817979000	-1.312406000	-0.144466000
C	-0.585688000	-0.129860000	-2.879469000
C	-1.670877000	0.607927000	-3.693293000
H	-1.374753000	1.659731000	-3.823342000
H	-1.805607000	0.180650000	-4.697403000
C	-2.965558000	0.535052000	-2.864224000
C	-3.684468000	1.882273000	-2.819350000
H	-3.029268000	2.679594000	-2.446684000
H	-4.583809000	1.837714000	-2.190102000
H	-3.999264000	2.149039000	-3.839506000
C	-3.952188000	-0.523309000	-3.370470000
H	-4.370449000	-0.183472000	-4.329354000
H	-4.787099000	-0.656103000	-2.668197000
H	-3.479390000	-1.497134000	-3.540988000
C	-0.511811000	-1.632894000	-3.249087000
H	-1.496574000	-2.117714000	-3.251610000
H	0.130400000	-2.176710000	-2.545062000
H	-0.087199000	-1.733843000	-4.259972000
C	0.808410000	0.467069000	-3.096231000
H	1.076650000	0.375488000	-4.161077000

H	1.555991000	-0.068079000	-2.497839000
H	0.851959000	1.526653000	-2.818409000
C	1.370582000	-1.342176000	-0.037178000
C	1.400676000	-2.810905000	-0.087016000
C	0.878458000	-3.577263000	0.975833000
H	0.485506000	-3.055871000	1.850521000
C	0.869710000	-4.971740000	0.936985000
H	0.467096000	-5.535360000	1.783635000
C	1.370291000	-5.650417000	-0.178980000
H	1.359215000	-6.742473000	-0.214814000
C	1.896123000	-4.910849000	-1.243111000
H	2.305175000	-5.426499000	-2.116401000
C	1.917954000	-3.516056000	-1.194146000
H	2.357530000	-2.949347000	-2.017662000
C	2.508426000	-0.587119000	-0.019309000
C	4.717204000	3.726797000	-0.774074000
H	4.815914000	4.530244000	-1.508812000
C	3.627724000	2.857107000	-0.842032000
H	2.877655000	2.983227000	-1.624502000
C	0.647903000	2.746025000	0.063548000
C	1.082132000	3.567422000	1.128730000
H	1.705055000	3.124123000	1.907714000
C	0.740214000	4.916885000	1.196926000
H	1.095348000	5.521575000	2.035961000
C	-0.046381000	5.503374000	0.198162000
H	-0.310157000	6.562429000	0.248915000
C	-0.485561000	4.712369000	-0.867551000
H	-1.096081000	5.152992000	-1.661037000
C	-0.151323000	3.357920000	-0.924766000
H	-0.502068000	2.755217000	-1.763366000
C	3.883205000	-1.158842000	-0.090617000
C	4.308910000	-2.146607000	0.813739000
H	3.623337000	-2.478344000	1.595002000
C	5.585076000	-2.705209000	0.722682000
H	5.893120000	-3.471878000	1.438628000
C	6.466224000	-2.288656000	-0.279048000
H	7.465762000	-2.724862000	-0.351301000

C	6.057698000	-1.306575000	-1.186603000
H	6.738133000	-0.971203000	-1.973759000
C	4.783427000	-0.746482000	-1.089743000
H	4.476370000	0.025718000	-1.797585000
C	-1.097827000	-0.565685000	2.576111000
C	-3.097814000	2.463873000	0.372039000
H	-2.379086000	2.478423000	-0.451922000
C	-2.298938000	2.751335000	1.653705000
H	-2.953913000	2.729877000	2.540843000
H	-1.835685000	3.745545000	1.592516000
H	-1.493436000	2.022855000	1.791725000
C	-4.119028000	3.588126000	0.144251000
H	-4.762514000	3.405203000	-0.727315000
H	-3.588687000	4.540060000	-0.013383000
H	-4.774808000	3.718868000	1.020036000
C	-4.789439000	-1.487212000	0.849365000
H	-5.187203000	-2.486519000	1.033333000
C	-4.409187000	-3.478700000	-1.362751000
H	-4.932933000	-3.970498000	-0.528042000
H	-3.978240000	-4.277229000	-1.986850000
H	-5.161190000	-2.943744000	-1.962349000
Be	-0.011765000	-0.191159000	-0.055144000
C	-1.379833000	-0.772278000	4.003483000
H	-1.168885000	-2.951708000	3.865170000
C	-0.748824000	0.408128000	4.773851000
H	-0.938565000	0.278135000	5.850216000
H	0.338083000	0.451466000	4.612056000
H	-1.184823000	1.365527000	4.453667000
C	-0.735384000	-2.110081000	4.425021000
H	0.350456000	-2.101226000	4.251452000
H	-0.916403000	-2.274793000	5.498068000
C	-2.905121000	-0.809260000	4.217832000
H	-3.368123000	-1.621918000	3.640774000
H	-3.113104000	-0.969706000	5.286613000
H	-3.371058000	0.134345000	3.903403000

Dianion [(CAAC)BeC$_4$Ph$_4$]$^{2-}$, singlet, UB3LYP-D3(BJ)/def2-SVP

Lowest frequency: 15.34 cm^{-1}

S^2: 0.0504

E+ZPE = –1927.288958 E$_h$

Be	0.052655000	-0.289718000	-0.868372000
C	-1.414284000	-1.247558000	-0.575486000
C	-2.436822000	-0.310234000	-0.314564000
C	-2.028018000	1.099241000	-0.462543000
C	-0.690504000	1.300590000	-0.837912000
C	1.661598000	-0.898777000	-0.981538000
N	2.756482000	-0.919685000	-0.071657000
C	3.604008000	-2.145582000	-0.197754000
C	3.053678000	-2.791288000	-1.494922000
H	2.430350000	-3.660675000	-1.237883000
H	3.870553000	-3.151109000	-2.145703000
C	2.157409000	-1.726051000	-2.164994000
C	5.106128000	-1.838076000	-0.303834000
H	5.675942000	-2.780107000	-0.373504000
H	5.459110000	-1.296062000	0.585901000
H	5.338209000	-1.236459000	-1.192497000
C	3.401709000	-3.094939000	0.995167000
H	3.974099000	-4.026888000	0.845881000
H	2.341720000	-3.360073000	1.097341000
H	3.738807000	-2.633124000	1.936330000
C	2.995466000	-0.827546000	-3.109072000
H	3.434268000	-1.413081000	-3.941501000
H	3.817573000	-0.330754000	-2.574456000
H	2.358307000	-0.036972000	-3.533498000
C	1.038399000	-2.359912000	-2.997409000
H	1.456996000	-2.908918000	-3.861138000
H	0.359186000	-1.582470000	-3.381404000
H	0.433420000	-3.052626000	-2.399747000
C	2.887422000	-0.105601000	1.092176000
C	2.093634000	-0.296917000	2.255591000
C	2.417705000	0.405533000	3.426907000
H	1.815738000	0.236868000	4.323361000
C	3.471440000	1.313649000	3.469676000

H	3.712693000	1.839926000	4.398597000
C	4.182181000	1.579399000	2.302556000
H	4.962261000	2.345581000	2.307640000
C	3.890064000	0.905840000	1.108937000
C	0.852945000	-1.174375000	2.276976000
H	0.760230000	-1.639800000	1.289506000
C	-0.398065000	-0.300800000	2.467264000
H	-1.307222000	-0.911864000	2.378079000
H	-0.458293000	0.474583000	1.690558000
H	-0.393695000	0.185552000	3.458978000
C	0.894710000	-2.278379000	3.343829000
H	0.025039000	-2.943853000	3.219841000
H	0.850887000	-1.859797000	4.364557000
H	1.804737000	-2.892323000	3.273786000
C	4.554371000	1.382127000	-0.173451000
H	4.324960000	0.637639000	-0.942542000
C	6.077827000	1.531588000	-0.077850000
H	6.497444000	1.796725000	-1.063398000
H	6.565165000	0.604536000	0.260786000
H	6.364635000	2.335990000	0.620751000
C	3.902917000	2.700212000	-0.615068000
H	4.291484000	3.023934000	-1.595808000
H	4.097943000	3.506145000	0.112357000
H	2.815856000	2.590010000	-0.709293000
C	-1.622940000	-2.672579000	-0.671551000
C	-0.618137000	-3.594786000	-0.240665000
H	0.295597000	-3.179868000	0.184041000
C	-0.759977000	-4.975363000	-0.355355000
H	0.048884000	-5.625178000	0.000116000
C	-1.914735000	-5.538993000	-0.918754000
H	-2.030144000	-6.623802000	-1.008393000
C	-2.916950000	-4.662140000	-1.370464000
H	-3.825407000	-5.069002000	-1.831195000
C	-2.780846000	-3.283518000	-1.251503000
H	-3.572707000	-2.635030000	-1.629402000
C	-3.802129000	-0.671186000	0.103793000
C	-4.030605000	-1.667318000	1.087427000

H	-3.164817000	-2.160073000	1.532313000
C	-5.314443000	-2.025256000	1.489954000
H	-5.440207000	-2.796556000	2.257863000
C	-6.441990000	-1.409217000	0.926291000
H	-7.451641000	-1.691925000	1.240916000
C	-6.243770000	-0.430541000	-0.057305000
H	-7.106918000	0.059776000	-0.521348000
C	-4.958433000	-0.073626000	-0.461275000
H	-4.828938000	0.680915000	-1.238396000
C	-2.949027000	2.216455000	-0.165191000
C	-3.668288000	2.286359000	1.052159000
H	-3.549723000	1.476262000	1.773501000
C	-4.511231000	3.357943000	1.343420000
H	-5.052284000	3.373046000	2.295818000
C	-4.667276000	4.412466000	0.434417000
H	-5.326437000	5.255676000	0.664370000
C	-3.954033000	4.369879000	-0.771664000
H	-4.055641000	5.185841000	-1.495454000
C	-3.113356000	3.296972000	-1.062650000
H	-2.561060000	3.275111000	-2.003617000
C	-0.073507000	2.605489000	-0.985934000
C	-0.152637000	3.649599000	-0.020178000
H	-0.715168000	3.464461000	0.896956000
C	0.468806000	4.881022000	-0.209395000
H	0.384615000	5.648122000	0.569035000
C	1.196673000	5.153128000	-1.378749000
H	1.677829000	6.124884000	-1.527510000
C	1.300849000	4.141971000	-2.343723000
H	1.875412000	4.320387000	-3.260081000
C	0.698277000	2.901012000	-2.145662000
H	0.797092000	2.113580000	-2.897078000

Dianion [(CAAC)BeC$_4$Ph$_4$]$^{2-}$, triplet, UB3LYP-D3(BJ)/def2-SVP			
**Lowest frequency: 11.46 cm^{-1}**			
**S^2: 2.0001**			
**E+ZPE = –1927.286567 E$_h$**			
Be	0.082780000	-0.172433000	-0.881394000
C	-1.289763000	-1.254306000	-0.557643000
C	-2.392456000	-0.408145000	-0.293892000
C	-2.097183000	1.032247000	-0.430611000
C	-0.805948000	1.343458000	-0.883971000
C	1.773648000	-0.567039000	-1.054993000
N	2.736544000	-0.831342000	-0.035526000
C	3.679178000	-1.938509000	-0.347548000
C	3.223223000	-2.349060000	-1.770587000
H	2.591791000	-3.247529000	-1.706901000
H	4.086350000	-2.598938000	-2.412956000
C	2.364894000	-1.181383000	-2.320973000
C	5.153553000	-1.493228000	-0.323511000
H	5.813681000	-2.353208000	-0.528451000
H	5.421913000	-1.087978000	0.664063000
H	5.358026000	-0.723075000	-1.077957000
C	3.532660000	-3.112729000	0.635105000
H	4.185316000	-3.951543000	0.335426000
H	2.497489000	-3.476565000	0.657254000
H	3.815910000	-2.809700000	1.655257000
C	3.242951000	-0.167309000	-3.095076000
H	3.667418000	-0.616697000	-4.015016000
H	4.083570000	0.199203000	-2.489275000
H	2.639916000	0.708541000	-3.379607000
C	1.277685000	-1.683147000	-3.282382000
H	1.722300000	-2.137939000	-4.187433000
H	0.638874000	-0.843947000	-3.602356000
H	0.627380000	-2.423867000	-2.797825000
C	2.832741000	-0.135560000	1.203435000
C	2.133194000	-0.577940000	2.359157000
C	2.440655000	-0.012922000	3.607424000
H	1.917828000	-0.377757000	4.495786000

C	3.364980000	1.019321000	3.729840000
H	3.597361000	1.442439000	4.712358000
C	3.951222000	1.544383000	2.578789000
H	4.623222000	2.403252000	2.658275000
C	3.682910000	0.999825000	1.315918000
C	0.954702000	-1.530433000	2.268574000
H	0.883785000	-1.869583000	1.230803000
C	-0.340976000	-0.754456000	2.563862000
H	-1.217399000	-1.397542000	2.404049000
H	-0.450583000	0.102981000	1.885442000
H	-0.352972000	-0.388749000	3.606269000
C	1.063299000	-2.760367000	3.178597000
H	0.222001000	-3.444115000	2.980994000
H	1.024473000	-2.482796000	4.246786000
H	1.997690000	-3.316533000	3.010816000
C	4.231361000	1.702867000	0.086789000
H	4.027017000	1.048690000	-0.765952000
C	5.740733000	1.973355000	0.145422000
H	6.088991000	2.397579000	-0.811925000
H	6.317395000	1.056750000	0.341964000
H	5.992754000	2.703860000	0.933038000
C	3.452261000	3.004795000	-0.148619000
H	3.763929000	3.486320000	-1.089621000
H	3.617821000	3.722315000	0.673182000
H	2.374905000	2.810978000	-0.220960000
C	-1.380042000	-2.676902000	-0.795221000
C	-0.227918000	-3.505255000	-0.613189000
H	0.690569000	-3.011430000	-0.300813000
C	-0.231081000	-4.877475000	-0.838704000
H	0.689828000	-5.448151000	-0.667919000
C	-1.390018000	-5.530399000	-1.291402000
H	-1.399257000	-6.609918000	-1.472257000
C	-2.531070000	-4.744324000	-1.525847000
H	-3.444061000	-5.216696000	-1.909025000
C	-2.531609000	-3.372765000	-1.292238000
H	-3.432512000	-2.801095000	-1.514509000
C	-3.732584000	-0.869879000	0.104680000

C	-3.894645000	-1.915245000	1.050698000
H	-2.997091000	-2.372367000	1.469506000
C	-5.150871000	-2.366307000	1.445058000
H	-5.223951000	-3.173340000	2.182447000
C	-6.317925000	-1.798340000	0.911660000
H	-7.306294000	-2.153017000	1.220796000
C	-6.186295000	-0.771667000	-0.033297000
H	-7.080702000	-0.316929000	-0.473654000
C	-4.927759000	-0.321397000	-0.428856000
H	-4.851771000	0.468225000	-1.177340000
C	-3.089500000	2.066487000	-0.064860000
C	-3.759659000	2.049498000	1.181001000
H	-3.547175000	1.233853000	1.874165000
C	-4.671592000	3.043436000	1.534199000
H	-5.171214000	2.994234000	2.507721000
C	-4.950441000	4.100429000	0.658444000
H	-5.664996000	4.881871000	0.936051000
C	-4.291849000	4.140594000	-0.578149000
H	-4.492374000	4.959471000	-1.277485000
C	-3.381248000	3.145701000	-0.929939000
H	-2.872345000	3.185536000	-1.894568000
C	-0.272281000	2.688697000	-1.009632000
C	-0.426251000	3.726663000	-0.046355000
H	-0.992606000	3.512887000	0.861726000
C	0.135850000	4.989048000	-0.223036000
H	-0.003272000	5.750078000	0.553521000
C	0.876152000	5.297655000	-1.374181000
H	1.313635000	6.291587000	-1.510726000
C	1.054979000	4.292774000	-2.336350000
H	1.642157000	4.499996000	-3.238508000
C	0.509807000	3.024836000	-2.151431000
H	0.668312000	2.241990000	-2.897343000

PhBC$_4$Ph$_4$, B3LYP/6-31+G*			
**Lowest frequency: 18.40 cm^{-1}**			
**E+ZPE = –1335.081224 E$_h$**			
C	1.260004000	0.389061000	-0.017913000
C	0.767310000	-0.882148000	-0.011183000
C	-0.767309000	-0.882148000	0.011008000
C	-1.260004000	0.389061000	0.017672000
B	-0.000002000	1.358109000	-0.000230000
C	0.000000000	2.908456000	-0.000066000
C	2.693046000	0.762868000	0.006211000
C	1.559912000	-2.134324000	-0.019869000
C	-1.559911000	-2.134323000	0.019834000
C	3.214772000	1.652150000	-0.952976000
C	4.562358000	2.018309000	-0.940497000
C	5.419444000	1.516768000	0.043515000
C	4.914431000	0.644060000	1.012570000
C	3.569609000	0.270402000	0.992234000
C	1.015589000	3.645721000	0.654067000
C	1.005712000	5.040611000	0.673907000
C	0.000009000	5.741643000	0.000223000
C	-1.005700000	5.040754000	-0.673603000
C	-1.015587000	3.645860000	-0.654045000
C	1.349897000	-3.136742000	0.945234000
C	2.122801000	-4.299145000	0.948754000
C	3.105818000	-4.494061000	-0.025753000
C	3.315632000	-3.512447000	-0.999348000
C	2.555314000	-2.342523000	-0.992371000
C	-1.349922000	-3.136834000	-0.945177000
C	-2.122827000	-4.299237000	-0.948565000
C	-3.105821000	-4.494056000	0.025986000
C	-3.315611000	-3.512346000	0.999490000
C	-2.555291000	-2.342424000	0.992380000
C	-2.693048000	0.762862000	-0.006379000
C	-3.569691000	0.270273000	-0.992268000
C	-4.914514000	0.643930000	-1.012542000
C	-5.419447000	1.516763000	-0.043556000
C	-4.562280000	2.018429000	0.940321000

C	-3.214693000	1.652269000	0.952736000
H	2.555531000	2.054435000	-1.717978000
H	4.941176000	2.700143000	-1.698312000
H	6.467374000	1.805757000	0.057896000
H	5.569200000	0.251780000	1.787218000
H	3.187653000	-0.408195000	1.750042000
H	1.816805000	3.117237000	1.161901000
H	1.788744000	5.581327000	1.199958000
H	0.000012000	6.829223000	0.000333000
H	-1.788729000	5.581582000	-1.199544000
H	-1.816807000	3.117485000	-1.161986000
H	0.584135000	-3.000132000	1.703450000
H	1.950960000	-5.056101000	1.710011000
H	3.701468000	-5.403466000	-0.027995000
H	4.075509000	-3.655213000	-1.763725000
H	2.728248000	-1.580185000	-1.746469000
H	-0.584179000	-3.000300000	-1.703426000
H	-1.951007000	-5.056268000	-1.709753000
H	-3.701472000	-5.403460000	0.028331000
H	-4.075470000	-3.655036000	1.763898000
H	-2.728205000	-1.580011000	1.746407000
H	-3.187798000	-0.408421000	-1.750021000
H	-5.569347000	0.251553000	-1.787086000
H	-6.467378000	1.805751000	-0.057889000
H	-4.941035000	2.700361000	1.698079000
H	-2.555388000	2.054653000	1.717631000

[(TMEDA)Mg$C_4Ph_2(SiMe_3)_2$], B3LYP/6-31+G*

Lowest frequency: 8.45 cm^{-1}

E+ZPE = –1981.473331 E_h

Si	-0.303542000	-3.377986000	0.222698000
Mg	-1.725564000	-0.001715000	-0.048072000
Si	-0.331609000	3.383754000	0.183072000
C	0.894829000	-0.768236000	-0.060283000
C	2.262415000	-1.409627000	-0.104938000
C	2.259348000	1.420768000	-0.047654000

C	0.892405000	0.776620000	-0.053273000
C	-0.244801000	1.533008000	-0.005802000
C	2.884851000	-1.703670000	-1.328869000
H	2.383130000	-1.434120000	-2.255626000
C	-0.239177000	-1.528600000	0.004566000
N	-3.338069000	0.018751000	-1.676180000
C	2.939579000	1.706530000	-1.242134000
H	2.484816000	1.428472000	-2.190189000
C	2.933875000	-1.767893000	1.075176000
H	2.476319000	-1.538862000	2.034775000
C	2.872581000	1.789037000	1.160959000
H	2.367760000	1.568168000	2.098752000
C	-4.595582000	0.350783000	-0.962601000
H	-5.473969000	0.104382000	-1.581876000
H	-4.612929000	1.433672000	-0.804979000
C	4.776446000	-2.703038000	-0.191413000
H	5.742073000	-3.201735000	-0.224897000
C	4.125093000	-2.343627000	-1.375231000
H	4.582552000	-2.564716000	-2.337268000
N	-3.579803000	-0.041110000	1.290879000
C	4.113172000	2.432412000	1.177164000
H	4.564939000	2.709217000	2.127400000
C	4.179547000	2.348736000	-1.232813000
H	4.682633000	2.563806000	-2.173159000
C	4.773178000	2.716879000	-0.021636000
H	5.738833000	3.216564000	-0.012383000
C	-2.007565000	-4.050303000	-0.338393000
H	-2.851497000	-3.532870000	0.138640000
H	-2.094822000	-5.114263000	-0.079934000
H	-2.136889000	-3.971917000	-1.425845000
C	-3.431596000	-1.302584000	-2.334617000
H	-2.468171000	-1.547570000	-2.788181000
H	-4.207949000	-1.296330000	-3.116919000
H	-3.664671000	-2.082894000	-1.609676000
C	4.174264000	-2.410771000	1.035633000
H	4.671757000	-2.680195000	1.964888000
C	-3.036760000	1.039915000	-2.703526000

H	-2.933509000	2.019969000	-2.235966000
H	-3.829821000	1.082001000	-3.467439000
H	-2.090406000	0.790797000	-3.190983000
C	0.996065000	4.492621000	-0.615123000
H	1.987080000	4.357841000	-0.170505000
H	0.711657000	5.547967000	-0.495560000
H	1.091045000	4.295884000	-1.690576000
C	-1.966485000	4.044126000	-0.567522000
H	-1.955714000	3.985672000	-1.664381000
H	-2.102428000	5.102414000	-0.306498000
H	-2.855065000	3.506456000	-0.210261000
C	-4.703565000	-0.376372000	0.380745000
H	-4.689736000	-1.458840000	0.220797000
H	-5.671137000	-0.135203000	0.850910000
C	-0.394035000	3.828494000	2.040124000
H	-1.196238000	3.293525000	2.565378000
H	-0.563001000	4.904629000	2.182969000
H	0.550056000	3.570533000	2.535426000
C	-3.441147000	-1.068828000	2.346412000
H	-3.243624000	-2.043294000	1.894903000
H	-4.351271000	-1.132879000	2.963967000
H	-2.595444000	-0.812935000	2.990134000
C	0.940024000	-4.485378000	-0.703189000
H	0.934246000	-4.283263000	-1.782041000
H	0.668627000	-5.541457000	-0.562720000
H	1.968002000	-4.351478000	-0.351960000
C	-3.786884000	1.276317000	1.931391000
H	-2.918142000	1.519288000	2.547595000
H	-4.687736000	1.268284000	2.566026000
H	-3.891931000	2.060339000	1.179438000
C	-0.178131000	-3.806660000	2.080099000
H	0.818294000	-3.569163000	2.471610000
H	-0.357615000	-4.877204000	2.250295000
H	-0.907794000	-3.246290000	2.679590000

**[MgC$_4$Ph$_2$(SiMe$_3$)$_2$], B3LYP/6-31+G***			
**Lowest frequency: 10.73 cm^{-1}**			
**E+ZPE = –1633.885232 E$_h$**			
Si	3.402352000	-1.641881000	-0.043437000
Mg	-0.000152000	-2.728553000	0.000237000
Si	-3.402388000	-1.642012000	0.043382000
C	0.784659000	-0.255938000	-0.004672000
C	1.427558000	1.103153000	0.105326000
C	-1.427498000	1.103262000	-0.105258000
C	-0.784654000	-0.255847000	0.004833000
C	-1.561364000	-1.366638000	0.086411000
C	2.117347000	1.658155000	-0.983860000
H	2.140485000	1.117977000	-1.927086000
C	1.561233000	-1.366855000	-0.086055000
C	-1.401386000	1.821316000	-1.311510000
H	-0.871118000	1.409656000	-2.166588000
C	1.401575000	1.821179000	1.311575000
H	0.871433000	1.409504000	2.166727000
C	-2.117437000	1.658222000	0.983834000
H	-2.140720000	1.118027000	1.927047000
C	2.740607000	3.590744000	0.337038000
H	3.246581000	4.548656000	0.427419000
C	2.765374000	2.891145000	-0.872414000
H	3.289847000	3.304002000	-1.731154000
C	-2.765470000	2.891224000	0.872336000
H	-3.290035000	3.304041000	1.731039000
C	-2.056503000	3.048638000	-1.429584000
H	-2.031380000	3.583176000	-2.376331000
C	-2.740570000	3.590832000	-0.337085000
H	-3.246532000	4.548743000	-0.427541000
C	3.682183000	-3.397351000	0.643315000
H	3.263870000	-3.504850000	1.653701000
H	4.752456000	-3.634166000	0.708041000
H	3.218489000	-4.162154000	0.004433000
C	2.056680000	3.048534000	1.429592000
H	2.031626000	3.583047000	2.376355000
C	-4.395624000	-0.457403000	-1.059061000

H	-4.351493000	0.577672000	-0.704701000
H	-5.450690000	-0.762818000	-1.084729000
H	-4.022571000	-0.469004000	-2.090943000
C	-3.681820000	-3.397226000	-0.644145000
H	-3.264282000	-3.503909000	-1.654937000
H	-4.752034000	-3.634522000	-0.708140000
H	-3.217259000	-4.162191000	-0.006089000
C	-4.111080000	-1.609045000	1.807833000
H	-3.550967000	-2.274082000	2.477814000
H	-5.159073000	-1.937302000	1.812508000
H	-4.076331000	-0.600187000	2.236039000
C	4.110861000	-1.608476000	-1.807951000
H	3.550955000	-2.273889000	-2.477751000
H	5.158954000	-1.936418000	-1.812593000
H	4.075768000	-0.599745000	-2.236394000
C	4.395593000	-0.457710000	1.059466000
H	4.350465000	0.577779000	0.706484000
H	5.450888000	-0.762462000	1.083864000
H	4.023417000	-0.470943000	2.091649000

$C_5H_5^+$, singlet, UB3LYP/6-31+G*

S^2: 0.2391

Lowest frequency: 383.87 cm^{-1}

C	0.000000000	0.000000000	1.209820000
C	0.000000000	1.154055000	0.377543000
C	0.000000000	0.719539000	-0.983905000
C	0.000000000	-0.719539000	-0.983905000
C	0.000000000	-1.154055000	0.377543000
H	0.000000000	1.359278000	-1.858663000
H	0.000000000	-2.182917000	0.720046000
H	0.000000000	2.182917000	0.720046000
H	0.000000000	-1.359278000	-1.858663000
H	0.000000000	0.000000000	2.294664000

$C_5H_7^+$, B3LYP/6-31+G*			
Lowest frequency: 199.39 cm^{-1}			
C	0.470378000	1.124938000	-0.000133000
C	1.290870000	0.000244000	0.000081000
C	0.470782000	-1.124760000	-0.000062000
C	-0.962083000	-0.768287000	-0.000006000
C	-0.962354000	0.767919000	0.000103000
H	0.832067000	-2.151339000	-0.000006000
H	-1.470606000	-1.213038000	0.870990000
H	-1.471114000	1.212586000	0.871025000
H	2.374828000	0.000309000	0.000239000
H	-1.470773000	-1.213055000	-0.870886000
H	-1.471261000	1.212589000	-0.870740000
H	0.831302000	2.151625000	-0.000515000

$C_5H_9^+$, B3LYP/6-31+G*			
Lowest frequency: 217.13 cm^{-1}			
C	-0.000058000	1.253591000	-0.000047000
C	1.194703000	0.443688000	0.093694000
C	0.746896000	-1.009418000	-0.199370000
C	-0.746780000	-1.009512000	0.199325000
C	-1.194759000	0.443576000	-0.093601000
H	1.415825000	0.563324000	1.189760000
H	1.339729000	-1.743275000	0.349666000
H	-0.872517000	-1.212574000	1.269239000
H	-2.088151000	0.853870000	0.394480000
H	0.872662000	-1.212383000	-1.269299000
H	2.088123000	0.854084000	-0.394253000
H	-1.339541000	-1.743388000	-0.349764000
H	-1.416044000	0.563209000	-1.189634000
H	-0.000094000	2.345585000	-0.000203000

C_4H_4BH, B3LYP/6-31+G*			
Lowest frequency: 193.00 cm^{-1}			
B	0.000000000	0.000000000	1.318405000
C	0.000000000	1.256133000	0.351446000
C	0.000000000	0.759135000	-0.901466000
C	0.000000000	-0.759135000	-0.901466000
C	0.000000000	-1.256133000	0.351446000
H	0.000000000	1.331280000	-1.828256000
H	0.000000000	-2.318277000	0.575984000
H	0.000000000	2.318277000	0.575984000
H	0.000000000	-1.331280000	-1.828256000
H	0.000000000	0.000000000	2.512754000

C_4H_6BH, B3LYP/6-31+G*			
Lowest frequency: 132.99 cm^{-1}			
B	0.105679000	1.350759000	-0.000151000
C	1.300032000	0.376064000	0.000277000
C	0.811860000	-0.891364000	-0.000105000
C	-0.690778000	-0.998878000	-0.000036000
C	-1.206585000	0.464338000	0.000149000
H	1.428869000	-1.792406000	-0.000619000
H	-1.034115000	-1.570991000	0.875131000
H	-1.843133000	0.677714000	0.872040000
H	2.369440000	0.577050000	0.000215000
H	-1.034525000	-1.571327000	-0.874826000
H	-1.842574000	0.677659000	-0.872191000
H	0.140471000	2.547542000	-0.000704000

C_4H_8BH, B3LYP/6-31+G*			
Lowest frequency: 201.93 cm^{-1}			
B	-0.000741000	1.380622000	0.000069000
C	-1.266633000	0.450922000	-0.111702000
C	-0.738576000	-0.967677000	0.225653000
C	0.739565000	-0.967051000	-0.225591000
C	1.266178000	0.452074000	0.111578000

H	-1.561641000	0.497005000	-1.179120000
H	-1.319610000	-1.775126000	-0.237066000
H	0.789479000	-1.122774000	-1.313179000
H	2.160865000	0.755132000	-0.447107000
H	-0.788303000	-1.123331000	1.313265000
H	-2.161757000	0.753004000	0.446806000
H	1.321336000	-1.773916000	0.237241000
H	1.561381000	0.498499000	1.178931000
H	-0.001253000	2.578785000	0.000255000

$C_4H_4BeH^-$, B3LYP/6-31+G*
Lowest frequency: 176.18 cm^{-1}

Be	0.000000000	0.000000000	1.541824000
C	0.000000000	1.352543000	0.350856000
C	0.000000000	0.749803000	-0.864400000
C	0.000000000	-0.749803000	-0.864400000
C	0.000000000	-1.352543000	0.350856000
H	0.000000000	1.267885000	-1.837004000
H	0.000000000	-2.450704000	0.358411000
H	0.000000000	2.450704000	0.358411000
H	0.000000000	-1.267885000	-1.837004000
H	0.000000000	0.000000000	2.952417000

$C_4H_6BeH^-$, B3LYP/6-31+G*
Lowest frequency: 89.63 cm^{-1}

Be	0.012815000	1.569903000	-0.063178000
C	1.378861000	0.454003000	0.020860000
C	0.880015000	-0.805579000	0.015109000
C	-0.628759000	-0.999032000	-0.054231000
C	-1.331051000	0.383743000	0.052541000
H	1.496412000	-1.721974000	0.044620000
H	-0.947178000	-1.725492000	0.721447000
H	-1.792319000	0.489708000	1.054041000
H	2.476670000	0.528555000	0.056726000
H	-0.879878000	-1.495036000	-1.011899000

H	-2.168885000	0.461342000	-0.662119000
H	-0.030478000	2.984474000	-0.155786000

$C_4H_8BeH^-$, B3LYP/6-31+G*

Lowest frequency: 160.34 cm^{-1}

Be	-0.000002000	1.583522000	-0.000008000
C	1.379215000	0.437785000	0.119229000
C	0.730287000	-0.915924000	-0.256404000
C	-0.730278000	-0.915939000	0.256379000
C	-1.379220000	0.437781000	-0.119194000
H	1.694176000	0.403231000	1.184544000
H	1.268083000	-1.817557000	0.104089000
H	-0.702075000	-1.006319000	1.357895000
H	-2.305283000	0.620367000	0.454899000
H	0.702089000	-1.006259000	-1.357924000
H	2.305311000	0.620381000	-0.454806000
H	-1.268069000	-1.817560000	-0.104149000
H	-1.694244000	0.403257000	-1.184490000
H	-0.000003000	3.004151000	-0.000085000

C_4H_4Be(CAAC), B3LYP/6-31+G*

Lowest frequency: 21.80 cm^{-1}

Be	-2.041554000	0.142267000	-0.902826000
C	-2.678048000	1.495341000	-1.830082000
C	-3.670500000	0.917470000	-2.550644000
C	-3.857262000	-0.556931000	-2.382650000
C	-3.019365000	-1.185155000	-1.521695000
C	-1.103556000	0.153411000	0.600251000
N	0.185434000	0.050982000	0.828754000
C	0.601615000	0.060584000	2.311144000
C	-0.719075000	0.506139000	2.972753000
H	-0.902270000	-0.041725000	3.902921000
H	-0.661926000	1.570663000	3.228545000
C	-1.843817000	0.277833000	1.928446000
C	1.746694000	1.041056000	2.582784000

H	2.652892000	0.773700000	2.030129000
H	1.985413000	1.008937000	3.652071000
H	1.472407000	2.068650000	2.336035000
C	1.042802000	-1.340568000	2.760524000
H	1.936126000	-1.666762000	2.221729000
H	0.258577000	-2.089332000	2.625069000
H	1.292124000	-1.305250000	3.827059000
C	-2.837116000	1.457645000	1.891259000
H	-2.331177000	2.400895000	1.656675000
H	-3.319888000	1.563028000	2.871199000
H	-3.615817000	1.302500000	1.137828000
C	-2.631753000	-1.031440000	2.186258000
H	-1.974787000	-1.906744000	2.231352000
H	-3.366921000	-1.206945000	1.395571000
H	-3.158523000	-0.953589000	3.145469000
C	1.176286000	-0.091586000	-0.238155000
C	1.513051000	-1.382121000	-0.717912000
C	2.531836000	-1.475970000	-1.677275000
H	2.808048000	-2.454343000	-2.059182000
C	3.178972000	-0.347909000	-2.168397000
H	3.967137000	-0.448830000	-2.910243000
C	2.785000000	0.912789000	-1.732701000
H	3.258278000	1.793063000	-2.157567000
C	1.776212000	1.076263000	-0.773837000
C	0.781884000	-2.669004000	-0.333483000
H	0.058928000	-2.437093000	0.451892000
C	-0.020185000	-3.213006000	-1.536326000
H	0.647057000	-3.500913000	-2.357747000
H	-0.733066000	-2.474089000	-1.912469000
H	-0.585563000	-4.104175000	-1.236521000
C	1.729355000	-3.764799000	0.198020000
H	2.401672000	-4.127560000	-0.588133000
H	1.145748000	-4.624683000	0.548415000
H	2.351585000	-3.416735000	1.028910000
C	1.333330000	2.501874000	-0.442209000
H	0.597273000	2.458416000	0.365262000
C	0.627104000	3.140844000	-1.657992000

H	1.321347000	3.258255000	-2.498937000
H	0.250937000	4.137004000	-1.393100000
H	-0.218822000	2.535394000	-1.993358000
C	2.497522000	3.402289000	0.020819000
H	3.206131000	3.589707000	-0.794164000
H	3.059095000	2.965883000	0.853088000
H	2.110332000	4.376522000	0.343370000
H	-2.544741000	2.575316000	-1.923020000
H	-4.341359000	1.456012000	-3.230031000
H	-4.654860000	-1.051731000	-2.949062000
H	-3.149122000	-2.259689000	-1.372444000

C_4H_6Be(CAAC), B3LYP/6-31+G*
Lowest frequency: 22.63 cm^{-1}

Be	-2.061690000	-0.070370000	-0.749029000
C	-2.433268000	0.469210000	-2.383970000
C	-3.797698000	-0.232482000	-2.650594000
C	-4.082202000	-1.233223000	-1.550323000
C	-3.255763000	-1.290498000	-0.478168000
C	-0.999412000	0.568422000	0.519776000
N	0.287102000	0.402485000	0.740550000
C	0.817262000	1.002397000	2.054989000
C	-0.353604000	1.935207000	2.423336000
H	-0.526415000	1.951576000	3.504443000
H	-0.116115000	2.960742000	2.115874000
C	-1.591240000	1.424627000	1.638048000
C	2.137006000	1.758346000	1.874405000
H	2.929975000	1.108034000	1.491359000
H	2.457909000	2.134627000	2.852542000
H	2.033174000	2.617417000	1.209485000
C	1.035624000	-0.105228000	3.098942000
H	1.826008000	-0.792877000	2.787664000
H	0.128131000	-0.681647000	3.293248000
H	1.348988000	0.356237000	4.042284000
C	-2.403098000	2.590681000	1.035397000
H	-1.794219000	3.195729000	0.353960000

H	-2.765627000	3.243212000	1.839940000
H	-3.269988000	2.223821000	0.476212000
C	-2.535052000	0.565246000	2.514400000
H	-2.017516000	-0.284802000	2.971169000
H	-3.363012000	0.169195000	1.920454000
H	-2.944218000	1.185713000	3.321609000
C	1.157236000	-0.317630000	-0.188021000
C	1.297349000	-1.723596000	-0.088136000
C	2.181590000	-2.355984000	-0.974924000
H	2.305365000	-3.433414000	-0.917121000
C	2.885164000	-1.642662000	-1.938199000
H	3.563325000	-2.157850000	-2.613825000
C	2.693613000	-0.269375000	-2.051126000
H	3.215914000	0.276995000	-2.831041000
C	1.829067000	0.423616000	-1.193890000
C	0.501627000	-2.610419000	0.870130000
H	-0.111757000	-1.969626000	1.507306000
C	-0.465765000	-3.531014000	0.094680000
H	0.082178000	-4.224517000	-0.554699000
H	-1.162824000	-2.955991000	-0.520948000
H	-1.056445000	-4.129528000	0.799338000
C	1.411511000	-3.461394000	1.781438000
H	1.959940000	-4.215364000	1.204714000
H	0.804929000	-3.995699000	2.522656000
H	2.150564000	-2.858048000	2.319145000
C	1.622932000	1.918575000	-1.445818000
H	0.997126000	2.321465000	-0.645185000
C	0.861779000	2.158088000	-2.766847000
H	1.437256000	1.792346000	-3.625955000
H	0.692345000	3.232592000	-2.911904000
H	-0.107708000	1.655394000	-2.767197000
C	2.949544000	2.707394000	-1.456090000
H	3.562134000	2.446523000	-2.326787000
H	3.552723000	2.523290000	-0.561155000
H	2.744559000	3.783385000	-1.515508000
H	-1.677397000	0.060213000	-3.076509000
H	-3.840496000	-0.730492000	-3.635105000

H	-4.971588000	-1.868177000	-1.665823000
H	-3.508763000	-2.017714000	0.301240000
H	-4.615760000	0.507845000	-2.677445000
H	-2.490847000	1.543660000	-2.612775000

$C_4H_8Be(CAAC)$, B3LYP/6-31+G*			
**Lowest frequency: 23.18 cm^{-1}**			
Be	-2.064866000	0.065843000	0.657366000
C	-2.354535000	0.257304000	2.384780000
C	-3.790503000	0.843589000	2.394369000
C	-3.973440000	1.710150000	1.132906000
C	-3.443946000	0.901190000	-0.075991000
C	-0.898817000	-0.874367000	-0.275933000
N	0.371863000	-0.646118000	-0.549392000
C	1.017975000	-1.617883000	-1.550532000
C	-0.008902000	-2.766139000	-1.517305000
H	-0.128362000	-3.225997000	-2.503811000
H	0.340177000	-3.548795000	-0.832853000
C	-1.334182000	-2.157406000	-0.987604000
C	2.417880000	-2.072099000	-1.126749000
H	3.108231000	-1.228267000	-1.028079000
H	2.814983000	-2.738790000	-1.900845000
H	2.404616000	-2.628848000	-0.188479000
C	1.126251000	-0.962742000	-2.937945000
H	1.825132000	-0.122618000	-2.923676000
H	0.162821000	-0.607180000	-3.310257000
H	1.510454000	-1.702149000	-3.649863000
C	-2.031670000	-3.100945000	0.014971000
H	-1.389681000	-3.319199000	0.875889000
H	-2.277082000	-4.050555000	-0.477545000
H	-2.962407000	-2.664234000	0.392494000
C	-2.322910000	-1.831921000	-2.133367000

H	-1.889826000	-1.153957000	-2.875527000
H	-3.231124000	-1.362438000	-1.748677000
H	-2.600200000	-2.761669000	-2.646006000
C	1.125419000	0.456890000	0.043276000
C	1.121594000	1.734659000	-0.568112000
C	1.913660000	2.738889000	0.008590000
H	1.927156000	3.725697000	-0.444897000
C	2.662771000	2.509247000	1.156422000
H	3.267567000	3.305076000	1.583818000
C	2.609703000	1.262537000	1.771372000
H	3.165205000	1.100302000	2.690405000
C	1.844521000	0.214989000	1.242594000
C	0.254709000	2.119749000	-1.766976000
H	-0.282790000	1.231397000	-2.106428000
C	-0.808649000	3.161936000	-1.357174000
H	-0.340811000	4.104985000	-1.050127000
H	-1.428229000	2.800997000	-0.532735000
H	-1.470525000	3.376756000	-2.204975000
C	1.081962000	2.663429000	-2.950942000
H	1.548772000	3.623881000	-2.702997000
H	0.430981000	2.830968000	-3.817558000
H	1.880974000	1.979137000	-3.254491000
C	1.791032000	-1.094770000	2.031615000
H	1.234243000	-1.829869000	1.444327000
C	1.023257000	-0.910516000	3.357747000
H	1.536284000	-0.197257000	4.014367000
H	0.960453000	-1.868135000	3.890149000
H	0.008494000	-0.546052000	3.185976000
C	3.192753000	-1.674238000	2.318601000
H	3.744613000	-1.048664000	3.029638000
H	3.803498000	-1.762761000	1.414396000
H	3.100520000	-2.670363000	2.768661000
H	-1.652590000	1.028890000	2.755994000
H	-4.029103000	1.413534000	3.308154000
H	-3.376253000	2.630261000	1.249187000
H	-3.373725000	1.506129000	-0.991114000
H	-4.518238000	0.016431000	2.352460000

H	-2.260762000	-0.590307000	3.079957000
H	-5.021119000	2.039091000	1.022700000
H	-4.181531000	0.104837000	-0.303024000

C_4H_4Be(CAAC)(*t*BuCN), B3LYP/6-31+G*
Lowest frequency: 11.45 cm^{-1}

N	1.681015000	-0.211358000	-0.055699000
N	-1.710930000	0.791085000	0.077939000
C	-1.043415000	4.095886000	-0.946273000
C	-0.647708000	2.902325000	-1.458482000
C	1.331593000	1.062900000	0.011155000
C	0.709025000	-1.297653000	-0.055251000
C	0.215164000	-1.786995000	-1.289600000
C	-0.671696000	-2.872497000	-1.257607000
H	-1.056797000	-3.269070000	-2.192977000
C	-1.078188000	-3.446156000	-0.056674000
H	-1.756690000	-4.296231000	-0.058045000
C	-0.555377000	-0.506485000	3.187351000
H	-0.886991000	0.329318000	2.566511000
H	-0.261230000	-0.105350000	4.165298000
H	-1.404460000	-1.183518000	3.345562000
C	0.636988000	-1.249249000	2.546305000
H	1.420387000	-0.505325000	2.398950000
C	0.263856000	-1.829761000	1.180624000
C	2.615305000	1.896955000	0.094574000
C	3.744214000	0.910720000	-0.305009000
H	3.997257000	1.054472000	-1.362391000
H	4.660222000	1.074052000	0.272936000
C	3.191054000	-0.509876000	-0.095614000
C	3.562728000	-1.450183000	-1.248473000
H	3.298521000	-1.030974000	-2.221353000

H	3.089984000	-2.432279000	-1.144683000
H	4.648555000	-1.600318000	-1.237869000
C	3.680880000	-1.150032000	1.213783000
H	4.758153000	-1.333543000	1.130042000
H	3.195179000	-2.113210000	1.394011000
H	3.522843000	-0.507191000	2.082362000
C	2.775305000	2.392984000	1.555918000
H	2.790856000	1.570149000	2.279382000
H	1.952180000	3.060818000	1.821106000
H	3.722598000	2.939841000	1.647472000
C	2.600557000	3.123924000	-0.833803000
H	3.570087000	3.636688000	-0.768815000
H	1.809725000	3.822362000	-0.554162000
H	2.434240000	2.835094000	-1.877030000
C	-0.620216000	3.136871000	1.234086000
C	-1.023347000	4.227477000	0.531068000
C	-2.798082000	0.395233000	0.105969000
C	0.531654000	-1.159155000	-2.648275000
H	1.288839000	-0.385702000	-2.501302000
C	-0.708645000	-0.452321000	-3.236202000
H	-1.524315000	-1.165538000	-3.410526000
H	-0.454046000	0.005624000	-4.200081000
H	-1.067850000	0.338488000	-2.573702000
C	1.080008000	-2.183156000	-3.664049000
H	1.934730000	-2.743366000	-3.271745000
H	1.400391000	-1.667874000	-4.577780000
H	0.312169000	-2.910040000	-3.954504000
C	-0.624854000	-2.913734000	1.146533000
H	-0.973445000	-3.342348000	2.082059000
C	1.175151000	-2.315894000	3.522381000
H	0.389746000	-3.017999000	3.826344000
H	1.548439000	-1.833316000	4.433726000
H	1.993111000	-2.901687000	3.089830000
Be	-0.299083000	1.870755000	-0.009324000
C	-4.215789000	-0.013107000	0.133117000
H	-4.846329000	1.871614000	1.066840000
C	-4.527938000	-0.826094000	-1.144635000

H	-5.584339000	-1.118306000	-1.133596000
H	-4.343409000	-0.232685000	-2.045795000
H	-3.917059000	-1.733022000	-1.195211000
C	-5.069333000	1.278344000	0.174692000
H	-4.884275000	1.903122000	-0.704427000
H	-6.130417000	1.003406000	0.192780000
C	-4.470593000	-0.870504000	1.394106000
H	-4.246586000	-0.308898000	2.306731000
H	-5.526409000	-1.163731000	1.419924000
H	-3.858542000	-1.778049000	1.385680000
H	-0.608743000	3.219078000	2.327784000
H	-1.331881000	5.180604000	0.980291000
H	-1.366832000	4.954672000	-1.549066000
H	-0.651619000	2.794461000	-2.549235000

C_4H_6Be(CAAC)(*t*BuCN), B3LYP/6-31+G*

Lowest frequency: 13.73 cm^{-1}

N	1.719172000	-0.115089000	-0.070074000
N	-1.703586000	0.645031000	0.113776000
C	-1.504186000	3.853216000	-1.080215000
C	-0.732559000	2.809352000	-1.452677000
C	1.300451000	1.134531000	0.069269000
C	0.815995000	-1.259192000	-0.090133000
C	0.329990000	-1.746115000	-1.328771000
C	-0.478023000	-2.892103000	-1.311740000
H	-0.854126000	-3.287652000	-2.251199000
C	-0.818534000	-3.527336000	-0.121548000
H	-1.435953000	-4.422669000	-0.135790000
C	-0.432366000	-0.642453000	3.190559000
H	-0.831044000	0.180190000	2.592011000
H	-0.147348000	-0.241309000	4.171240000
H	-1.231004000	-1.378180000	3.349472000
C	0.793864000	-1.290740000	2.512343000
H	1.525935000	-0.494860000	2.372863000
C	0.433716000	-1.857036000	1.137211000
C	2.544443000	2.027423000	0.183311000

C	3.713598000	1.129393000	-0.297041000
H	3.930447000	1.344718000	-1.350274000
H	4.634624000	1.310757000	0.267639000
C	3.241557000	-0.327963000	-0.155713000
C	3.639635000	-1.178115000	-1.368122000
H	3.325785000	-0.722431000	-2.309299000
H	3.229991000	-2.191557000	-1.309513000
H	4.732521000	-1.261408000	-1.389761000
C	3.795203000	-1.017058000	1.102859000
H	4.877991000	-1.139838000	0.985726000
H	3.362455000	-2.012985000	1.234289000
H	3.626092000	-0.437596000	2.012685000
C	2.715096000	2.425523000	1.673121000
H	2.819941000	1.555144000	2.329421000
H	1.859109000	3.006994000	2.021566000
H	3.620064000	3.037222000	1.780005000
C	2.457429000	3.311289000	-0.660290000
H	3.406209000	3.859704000	-0.580942000
H	1.645306000	3.958692000	-0.324727000
H	2.277534000	3.084749000	-1.716230000
C	-0.884602000	3.127284000	1.231954000
C	-1.838100000	4.022552000	0.390033000
C	-2.768417000	0.190802000	0.153824000
C	0.568766000	-1.061067000	-2.675886000
H	1.260866000	-0.230109000	-2.520856000
C	-0.736842000	-0.448503000	-3.227107000
H	-1.491781000	-1.224356000	-3.407617000
H	-0.538054000	0.052025000	-4.182859000
H	-1.149850000	0.293552000	-2.540561000
C	1.178323000	-2.011519000	-3.728213000
H	2.082379000	-2.511960000	-3.367791000
H	1.438002000	-1.448843000	-4.633245000
H	0.464700000	-2.789609000	-4.024427000
C	-0.378686000	-2.998544000	1.088262000
H	-0.677181000	-3.476739000	2.017075000
C	1.416014000	-2.344611000	3.451458000
H	0.684008000	-3.105535000	3.747023000

H	1.770268000	-1.862325000	4.370596000
H	2.264541000	-2.862332000	2.991939000
Be	-0.367428000	1.868146000	0.029147000
C	-4.156178000	-0.311409000	0.190846000
H	-3.653141000	-2.087086000	1.375684000
C	-5.099559000	0.912165000	0.289497000
H	-6.138173000	0.561645000	0.306819000
H	-4.910388000	1.485343000	1.202449000
H	-4.971001000	1.580916000	-0.567128000
C	-4.327813000	-1.226516000	1.425036000
H	-4.127917000	-0.681570000	2.353300000
H	-5.359632000	-1.595004000	1.456857000
C	-4.434593000	-1.101881000	-1.108922000
H	-3.761304000	-1.959952000	-1.201023000
H	-5.467068000	-1.469615000	-1.090183000
H	-4.309913000	-0.467096000	-1.991924000
H	-0.027000000	3.744712000	1.545364000
H	-1.802846000	5.089376000	0.677669000
H	-1.927078000	4.582343000	-1.786648000
H	-0.524991000	2.711796000	-2.526556000
H	-1.371919000	2.804601000	2.167519000
H	-2.891159000	3.727729000	0.557595000

C_4H_8Be(CAAC)(*t*BuCN), B3LYP/6-31+G*

Lowest frequency: 14.70 cm^{-1}

N	1.694240000	-0.235207000	-0.051029000
N	-1.669317000	0.674306000	0.008402000
C	-1.575160000	4.037540000	-0.723398000
C	-0.821478000	2.906973000	-1.467286000
C	1.343419000	1.045509000	-0.103712000
C	0.729075000	-1.323537000	0.018924000
C	0.250853000	-1.891764000	-1.188339000
C	-0.626011000	-2.981584000	-1.100107000
H	-0.998825000	-3.437251000	-2.013311000
C	-1.034032000	-3.489670000	0.129967000
H	-1.703155000	-4.346118000	0.173753000

C	-0.560256000	-0.394304000	3.225637000
H	-0.912215000	0.403512000	2.568021000
H	-0.272242000	0.059789000	4.181977000
H	-1.391163000	-1.084409000	3.420380000
C	0.647842000	-1.136150000	2.614345000
H	1.412040000	-0.380853000	2.427247000
C	0.285091000	-1.791204000	1.280258000
C	2.646193000	1.869485000	-0.147484000
C	3.750818000	0.830580000	-0.473150000
H	3.952633000	0.838287000	-1.551443000
H	4.694363000	1.056608000	0.035106000
C	3.201140000	-0.543769000	-0.065496000
C	3.568144000	-1.637278000	-1.076627000
H	3.312811000	-1.354548000	-2.099414000
H	3.086097000	-2.591968000	-0.842226000
H	4.652523000	-1.793770000	-1.039531000
C	3.705839000	-0.993639000	1.316317000
H	4.787979000	-1.157367000	1.256015000
H	3.246499000	-1.937589000	1.621013000
H	3.527032000	-0.248263000	2.094063000
C	2.880943000	2.537598000	1.230647000
H	2.870397000	1.817293000	2.055211000
H	2.111143000	3.283290000	1.435159000
H	3.859370000	3.035311000	1.227189000
C	2.633033000	2.974538000	-1.219034000
H	3.634187000	3.421790000	-1.284737000
H	1.919379000	3.763444000	-0.975525000
H	2.371221000	2.577562000	-2.205881000
C	-0.622321000	2.985181000	1.331468000
C	-0.844842000	4.328718000	0.599362000
C	-2.750925000	0.258787000	0.028393000
C	0.586119000	-1.349193000	-2.579292000
H	1.362994000	-0.588759000	-2.472352000
C	-0.631050000	-0.643869000	-3.214369000
H	-1.470078000	-1.340270000	-3.339148000
H	-0.364109000	-0.261295000	-4.207486000
H	-0.963430000	0.200798000	-2.606675000

C	1.113843000	-2.442003000	-3.532532000
H	1.943803000	-3.008668000	-3.098003000
H	1.463697000	-1.985514000	-4.466536000
H	0.326849000	-3.157801000	-3.797990000
C	-0.591286000	-2.886046000	1.302789000
H	-0.938530000	-3.266090000	2.259620000
C	1.211442000	-2.143523000	3.638603000
H	0.438643000	-2.840587000	3.984024000
H	1.583968000	-1.609755000	4.521272000
H	2.034407000	-2.740085000	3.230918000
Be	-0.297726000	1.839205000	-0.068824000
C	-4.156680000	-0.193401000	0.045682000
H	-3.759656000	-1.908055000	1.353738000
C	-5.058657000	1.064901000	0.038176000
H	-6.108959000	0.750896000	0.047334000
H	-4.875058000	1.689523000	0.917951000
H	-4.884580000	1.672474000	-0.855276000
C	-4.403987000	-1.023840000	1.326290000
H	-4.215461000	-0.429359000	2.226031000
H	-5.449203000	-1.353721000	1.343239000
C	-4.422369000	-1.052294000	-1.212427000
H	-3.778057000	-1.936832000	-1.229590000
H	-5.467494000	-1.382815000	-1.206167000
H	-4.247591000	-0.477950000	-2.127819000
H	0.087556000	3.094431000	2.165957000
H	0.130051000	4.789801000	0.364002000
H	-1.699010000	4.957638000	-1.322612000
H	-1.473396000	2.438887000	-2.224332000
H	-1.575938000	2.681322000	1.801195000
H	-1.391507000	5.078050000	1.200943000
H	-2.597121000	3.699044000	-0.479528000
H	0.015146000	3.342960000	-2.033718000

References

[1] V. Lavallo, Y. Canac, C. Präsang, B. Donnadieu, G. Bertrand, *Angew. Chem. Int. Ed.* **2005**, *44*, 5705–5709.

[2] R. Jazzar, R. D. Dewhurst, J.-B. Bourg, B. Donnadieu, Y. Canac, G. Bertrand, *Angew. Chem. Int. Ed.* **2007**, *46*, 5899–5902.

[3] W. Schlenk, E. Bergmann, *Justus Liebigs Ann. Chem.* **1928**, *463*, 1–97.

[4] G. Sheldrick, *Acta Cryst.* **2015**, *A71*, 3–8.

[5] G. Sheldrick, *Acta Cryst.* **2008**, *A64*, 112–122.

[6] S. H. Vosko, L. Wilk, M. Nusair, *Can. J. Phys.* **1980**, *58*, 1200–1211.

[7] C. Lee, W. Yang, R. G. Parr, *Phys. Rev. B* **1988**, *37*, 785–789.

[8] A. D. Becke, *J. Chem. Phys.* **1993**, *98*, 5648–5652.

[9] P. J. Stephens, F. J. Devlin, C. F. Chabalowski, M. J. Frisch, *J. Phys. Chem.* **1994**, *98*, 11623–11627.

[10] F. Weigend, R. Ahlrichs, *Phys. Chem. Chem. Phys.* **2005**, *7*, 3297.

[11] S. Grimme, J. Antony, S. Ehrlich, H. Krieg, *J. Chem. Phys.* **2010**, *132*, 154104.

[12] S. Grimme, S. Ehrlich, L. Goerigk, *J. Comput. Chem.* **2011**, *32*, 1456–1465.

[13] R. Seeger, J. A. Pople, *J. Chem. Phys.* **1977**, *66*, 3045–3050.

[14] R. Bauernschmitt, R. Ahlrichs, *J. Chem. Phys.* **1996**, *104*, 9047–9052.

[15] B. O. Roos, in *Adv. Chem. Phys. Ab Initio Methods Quantum Chem. Part 2, Vol. 69* (Ed.: K. P. Lawley), John Wiley & Sons, **1987**, pp. 399–445.

[16] C. Angeli, R. Cimiraglia, J.-P. Malrieu, *Chem. Phys. Lett.* **2001**, *350*, 297–305.

[17] C. Angeli, R. Cimiraglia, S. Evangelisti, T. Leininger, J.-P. Malrieu, *J. Chem. Phys.* **2001**, *114*, 10252–10264.

[18] C. Angeli, R. Cimiraglia, J.-P. Malrieu, *J. Chem. Phys.* **2002**, *117*, 9138–9153.

[19] J. Zheng, X. Xu, D. G. Truhlar, *Theor. Chem. Acc.* **2011**, *128*, 295–305.

[20] K. Eichkorn, O. Treutler, H. Öhm, M. Häser, R. Ahlrichs, *Chem. Phys. Lett.* **1995**, *240*, 283–290.

[21] K. Eichkorn, F. Weigend, O. Treutler, R. Ahlrichs, *Theor. Chem. Acc.* **1997**, *97*, 119–124.

[22] F. Neese, F. Wennmohs, A. Hansen, U. Becker, *Chem. Phys.* **2009**, *356*, 98–109.

[23] R. Izsák, F. Neese, *J. Chem. Phys.* **2011**, *135*, 144105.

[24] Y. Guo, K. Sivalingam, E. F. Valeev, F. Neese, *J. Chem. Phys.* **2016**, *144*, 094111.

[25] K. Yamaguchi, *Chem. Phys. Lett.* **1975**, *33*, 330–335.

[26] S. Yamanaka, M. Okumura, M. Nakano, K. Yamaguchi, *J. Mol. Struct.* **1994**, *310*, 205–218.

[27] M. Nakano, *Top. Curr. Chem.* **2017**, *375*, 47.

[28] D. Doehnert, J. Koutecky, *J. Am. Chem. Soc.* **1980**, *102*, 1789–1796.

[29] J. Michl, V. Bonačić-Koutecký, *Tetrahedron* **1988**, *44*, 7559–7585.

[30] M. P. Mitoraj, A. Michalak, T. Ziegler, *J. Chem. Theory Comput.* **2009**, *5*, 962–975.

[31] L. Zhao, M. von Hopffgarten, D. M. Andrada, G. Frenking, *WIREs Comput. Mol. Sci.* **2018**, *8*, e1345.

[32] P. v. R. Schleyer, C. Maerker, A. Dransfeld, H. Jiao, N. J. R. van Eikema Hommes, *J. Am. Chem. Soc.* **1996**, *118*, 6317–6318.

[33] Z. Chen, C. S. Wannere, C. Corminboeuf, R. Puchta, P. v. R. Schleyer, *Chem. Rev.* **2005**, *105*, 3842–3888.

[34] R. Ditchfield, *Mol. Phys.* **1974**, *27*, 789–807.

[35] K. Wolinski, J. F. Hinton, P. Pulay, *J. Am. Chem. Soc.* **1990**, *112*, 8251–8260.

[36] J. R. Cheeseman, G. W. Trucks, T. A. Keith, M. J. Frisch, *J. Chem. Phys.* **1996**, *104*, 5497–5509.

[37] A. Stanger, *J. Org. Chem.* **2006**, *71*, 883–893.

[38] A. C. Tsipis, *Phys. Chem. Chem. Phys.* **2009**, *11*, 8244.

[39] P. v. R. Schleyer, H. Jiao, B. Goldfuss, P. K. Freeman, *Angew. Chemie Int. Ed. Engl.* **1995**, *34*, 337–340.

[40] M. K. Cyrański, P. v. R. Schleyer, T. M. Krygowski, H. Jiao, G. Hohlneicher, *Tetrahedron* **2003**, *59*, 1657–1665.

[41] J. A. Montgomery, M. J. Frisch, J. W. Ochterski, G. A. Petersson, *J. Chem. Phys.* **1999**, *110*, 2822–2827.

[42] J. A. Montgomery, M. J. Frisch, J. W. Ochterski, G. A. Petersson, *J. Chem. Phys.* **2000**, *112*, 6532–6542.

[43] M. J. Frisch, G. W. Trucks, H. B. Schlegel, G. E. Scuseria, M. A. Robb, J. R. Cheeseman, G. Scalmani, V. Barone, B. Mennucci, G. A. Petersson, H. Nakatsuji, M. Caricato, X. Li, H. P. Hratchian, A. F. Izmaylov, J. Bloino, G. Zheng, J. L. Sonnenberg, M. Hada, M. Ehara, K. Toyota, R. Fukuda, J. Hasegawa, M. Ishida, T. Nakajima, Y. Honda, O. Kitao, H. Nakai, T. Vreven, J. A. Montgomery Jr., J. E. Peralta, F. Ogliaro, M. Bearpark, J. J. Heyd, E. Brothers, K. N. Kudin, V. N. Staroverov, R. Kobayashi, J. Normand, K. Raghavachari, A. Rendell, J. C. Burant, S. S. Iyengar, J. Tomasi, M. Cossi, N. Rega, J. M. Millam, M. Klene, J. E. Knox, J. B. Cross, V. Bakken, C. Adamo, J. Jaramillo, R. Gomperts, R. E. Stratmann, O. Yazyev, A. J. Austin, R. Cammi, C. Pomelli, J. W. Ochterski, R. L. Martin, K. Morokuma, V. G. Zakrzewski, G. A. Voth, P. Salvador, J. J. Dannenberg, S. Dapprich, A. D. Daniels, Ö. Farkas, J. B. Foresman, J. V Ortiz, J. Cioslowski, D. J. Fox, Gaussian 16, Revision B.01, Gaussian, Inc., Wallingford CT, **2016**.

[44] F. Neese, *WIREs Comput. Mol. Sci.* **2012**, *2*, 73–78.

[45] G. te Velde, F. M. Bickelhaupt, E. J. Baerends, C. Fonseca Guerra, S. J. A. van Gisbergen, J. G. Snijders, T. Ziegler, *J. Comput. Chem.* **2001**, *22*, 931–967.

[46] G. A. Zhurko, *ChemCraft* Version 1.8, **2020**, http://www.chemcraftprog.com

[47] R. Herges, D. Geuenich, *J. Phys. Chem. A* **2001**, *105*, 3214–3220.

[48] D. Geuenich, K. Hess, F. Köhler, R. Herges, *Chem. Rev.* **2005**, *105*, 3758–3772.

[49] T. A. Keith, R. F. W. Bader, *Chem. Phys. Lett.* **1992**, *194*, 1–8.

[50] T. A. Keith, R. F. W. Bader, *Chem. Phys. Lett.* **1993**, *210*, 223–231.
